# The Potent Cdc7-Dbf4 (DDK) Kinase Inhibitor XL413 Has Limited Activity in Many Cancer Cell Lines and Discovery of Potential New DDK Inhibitor Scaffolds

**DOI:** 10.1371/journal.pone.0113300

**Published:** 2014-11-20

**Authors:** Nanda Kumar Sasi, Kanchan Tiwari, Fen-Fen Soon, Dorine Bonte, Tong Wang, Karsten Melcher, H. Eric Xu, Michael Weinreich

**Affiliations:** 1 Laboratory of Genome Integrity and Tumorigenesis, Van Andel Research Institute (VARI), Grand Rapids, MI, United States of America; 2 Graduate Program in Genetics, Michigan State University, East Lansing, MI, United States of America; 3 Laboratory of Structural Sciences, VARI, Grand Rapids, MI, United States of America; 4 Translational Drug Development, Inc. (TD2), Scottsdale, AZ, United States of America; 5 Laboratory of Structural Biology and Biochemistry, VARI, Grand Rapids, MI, United States of America; Columbia University, United States of America

## Abstract

Cdc7-Dbf4 kinase or DDK (Dbf4-dependent kinase) is required to initiate DNA replication by phosphorylating and activating the replicative Mcm2-7 DNA helicase. DDK is overexpressed in many tumor cells and is an emerging chemotherapeutic target since DDK inhibition causes apoptosis of diverse cancer cell types but not of normal cells. PHA-767491 and XL413 are among a number of potent DDK inhibitors with low nanomolar IC_50_ values against the purified kinase. Although XL413 is highly selective for DDK, its activity has not been extensively characterized on cell lines. We measured anti-proliferative and apoptotic effects of XL413 on a panel of tumor cell lines compared to PHA-767491, whose activity is well characterized. Both compounds were effective biochemical DDK inhibitors but surprisingly, their activities in cell lines were highly divergent. Unlike PHA-767491, XL413 had significant anti-proliferative activity against only one of the ten cell lines tested. Since XL413 did not effectively inhibit DDK in multiple cell lines, this compound likely has limited bioavailability. To identify potential leads for additional DDK inhibitors, we also tested the cross-reactivity of ∼400 known kinase inhibitors against DDK using a DDK thermal stability shift assay (TSA). We identified 11 compounds that significantly stabilized DDK. Several inhibited DDK with comparable potency to PHA-767491, including Chk1 and PKR kinase inhibitors, but had divergent chemical scaffolds from known DDK inhibitors. Taken together, these data show that several well-known kinase inhibitors cross-react with DDK and also highlight the opportunity to design additional specific, biologically active DDK inhibitors for use as chemotherapeutic agents.

## Introduction

The initiation of DNA replication is temporally divided into two phases during the cell cycle. First, an inactive form of the replicative MCM (mini-chromosome maintenance) helicase is loaded onto origin DNA in G1 phase and then activated upon entry into and during S phase by two sets of kinases: cyclin-dependent kinase and Dbf4-dependent kinase (DDK) [1]. DDK is a two-subunit Ser/Thr kinase composed of the Cdc7 kinase and Dbf4 regulatory subunits. DDK mediated phosphorylation of the six-subunit Mcm2-7 (MCM) helicase is thought to bring about a conformational change in its structure leading to helicase activation [2], [3]. MCM activation is followed by localized DNA unwinding, recruitment of the replisome machinery and the initiation of bi-directional DNA synthesis [1]. Other functions of DDK include facilitation of chromosomal segregation in mitosis and meiosis [4], [5], the initiation of meiotic recombination [6], [7], and activation of DNA repair pathways including trans-lesion DNA repair [8], [9].

Cdc7 kinase activity depends on association with its regulatory subunit, Dbf4 [10], [11]. Dbf4 is a cell cycle regulated protein whose abundance peaks during S-phase and then is degraded by end of mitosis [12]–[14]. Interaction with Dbf4 is necessary for Cdc7 ATP binding and substrate recognition [15]. Like all protein kinases, the DDK crystal structure reveals an active site in a deep cleft between the N- and C-terminal lobes [16], [17]. The Dbf4 Zn-finger (“motif C”) binds to the N-terminal lobe of DDK and is necessary for human DDK activity but is not essential for budding or fission yeast DDK kinase activity [18]–[20]. Dbf4 motif M enhances its association with the Cdc7 subunit and is required for the full activity of the kinase in yeast and humans [16], [18], [19], [21]. DDK phosphorylates multiple subunits of the MCM helicase [22]–[24] and a recent study in budding yeast indicates that Cdc7 and Dbf4 physically interact with distinct subunits of the Mcm2-7 complex [25].

**Figure 1:**
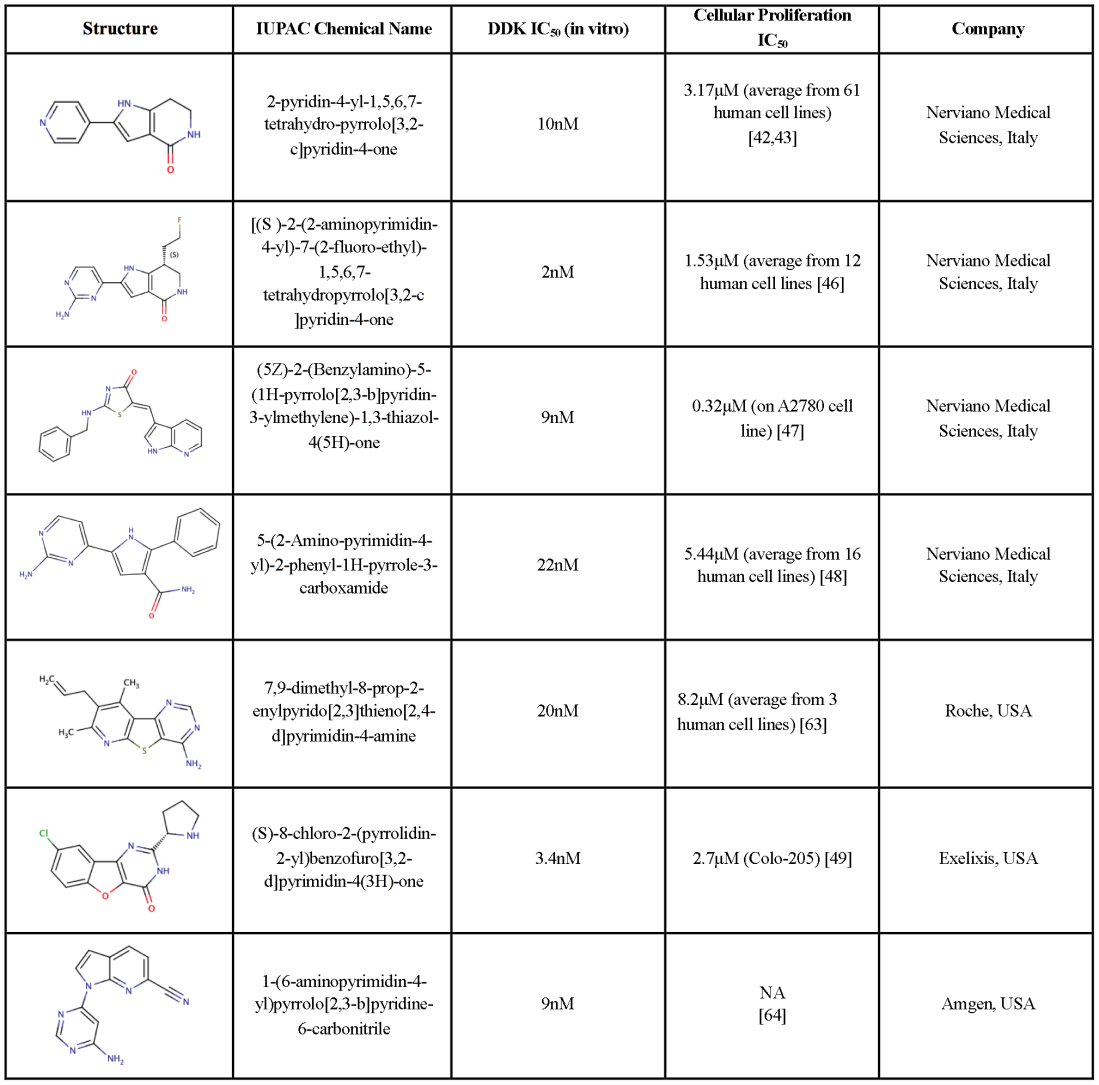
DDK inhibitors synthesized by various pharmaceutical companies.

DDK is over expressed in a number of primary tumors and tumor cell lines [26]–[32]. DDK over expression has also been associated with poor prognosis in breast cancers [33], advanced clinical stage in ovarian carcinoma [34], and with aggressive phenotype in papillary thyroid carcinomas [35]. Regulating the levels of DDK in tumor cells is an attractive tumor therapeutic strategy. Using neutralizing antibodies, Hunter and colleagues were the first to show that DDK depletion leads to severe disruption of DNA replication in HeLa cells [10]. Using small interfering RNAs, Santocanale and colleagues further showed that DDK depletion led to p53-independent apoptosis in HeLa cells whereas a normal human dermal fibroblast cell line underwent a reversible cell-cycle arrest [36]. HeLa cells were unable to arrest at the G1-S phase transition, progressing through a lethal S phase resulting in cell death via apoptosis. This finding has been corroborated in a number of different cell lines [37]–[39]. Importantly, tumor cell death induced by depletion of DDK is not accompanied by the induction of known checkpoint markers. Similar cellular responses are seen upon depletion of other components of the replication initiation machinery, including the Cdc6, Cdc45 and Mcm2 subunits [40], [41]. The tumor cell specific killing observed by the depletion of DDK has aroused interest as a pharmaceutical target for cancer therapy. Efforts by multiple pharmaceutical companies have led to a number of small molecule DDK inhibitors (Figure 1).

The first well-characterized DDK inhibitor was a pyrrolopyridinone molecule (PHA-767491, Figure 1) [42], [43]. It is a potent DDK inhibitor with an IC_50_ of 10 nM using purified kinase. PHA-767491 is also an effective cell growth inhibitor, with an average IC50  = 3.14 µM among 61 tumor cell lines [43]. PHA-767491 also inhibits purified Cdk9 with an IC_50_ of 34 nM but is a much less potent inhibitor of many other kinases tested [43]. Hence PHA-767491 is a dual DDK/Cdk9 inhibitor. Recent studies have suggested that inhibition of Cdk9, a kinase that targets RNA Polymerase II, might enhance the apoptotic response induced by PHA-767491 in some cell lines [43]–[45]. Modifications of this compound led to the identification of several other potent inhibitors of DDK with some exhibiting superior selectivity and sensitivity [46]–[48]. XL413, a structurally distinct DDK inhibitor, is a benzofuropyrimidinone based compound with a reported IC_50_ of 3.4 nM against purified DDK and inhibits cell-proliferation of Colo-205 cells with an IC_50_ of 2.69 µM [49]. It was also highly selective for DDK when tested against a panel of 100 kinases [49].

The increased activity and selectivity of XL413 over PHA-767491 was rationalized by the crystal structure of DDK in complex with the two DDK inhibitors [16]. One reason XL413 might be a more specific inhibitor is that it made contacts with three of the most variant residues in the kinase active site when compared to PHA-767491, which interacted with two of these residues. It was therefore unexpected to find that XL413 was not a particularly potent cell growth inhibitor in most of the cell lines we tested, since Cdc7 is essential for cell cycle progression. XL413 inhibited proliferation and induced apoptosis in Colo-205 cells as shown previously [49] but had limited activity in 9 other tumor cell lines tested. Although both compounds are comparable biochemical DDK inhibitors, PHA-767491 exhibited superior activity to XL413 in cell lines. Analysis of DDK-specific Mcm2 phosphorylation levels suggests that XL413 might have poor bioavailability in these and other cancer cell lines. To aid in the development of additional DDK inhibitors, we tested whether known protein kinase inhibitors (i.e., those not designed to inhibit DDK) exhibited cross-reaction with DDK. We screened ∼400 compounds using a thermal stability shift assay (TSA) and identified 12 molecules that shifted the thermal stability of DDK, several with divergent chemical scaffolds and with nearly equivalent potency as PHA-767491. These compounds are therefore unlikely to be highly specific for a single target. Our data highlight the opportunity to design additional specific, biologically active DDK inhibitors for use as chemotherapeutic agents.

## Materials and Methods

### Synthesis of PHA-767491 and XL413

The DDK inhibitors, PHA-767491 and XL413, were synthesized as described previously [42], [49]. HPLC analysis and mass spectrometry were performed on both compounds, which confirmed the correct molecular mass and a high level of purity (>99%) for both.

### Cell lines

HeLa cells (ATCC) were cultured in MEM supplemented with Earle's salts, 2 mM glutamine, 10% heat-inactivated fetal bovine serum (HI FBS), 1.5 g/L sodium bicarbonate, 0.1 mM non-essential amino acids, 1 mM sodium pyruvate, 50 units/ml of penicillin, and 50 µg/ml of streptomycin. MDA-MB-453 (ATCC) cells were grown in DMEM supplemented with 4.5 g/L D-glucose, 4 mM L-glutamine, 110 mg/L sodium pyruvate, 10% HI FBS, 50 units/ml of penicillin, and 50 µg/ml of streptomycin. HCC1954 (ATCC), HCC1187 (ATCC), BT-549 (NCI-60), MCF-7 (NCI-60), and Colo-205 (NCI-60) cells were all cultured in RPMI 1640 media supplemented with 10% HI FBS, 50 units/ml of penicillin, and 50 µg/ml of streptomycin. HCT-116 p53^+/+^ and p53^-/-^ cell lines were cultured in McCoy's 5A medium supplemented with 10% HI FBS, 50 units/ml of penicillin, and 50 µg/ml of streptomycin. All cells were maintained at 37°C with 5% CO_2_ in a humidified incubator.

### DDK protein induction

pKT37 is a pETDuet-1 (Novagen)-vector that co-expresses His6-Smt3-HsCdc7 (codon optimized, Genescript) and Dbf4 residues 341–674, which contains motifs M and C required to bind and activate Cdc7. *E. coli* BL21-RIPL was transformed with pKT37 and a fresh colony was grown overnight in LB containing 150 µg/ml ampicillin, 50 µg/ml chloramphenicol and 1% glucose. Two liters of LB containing 150 µg/ml ampicillin and 50 µg/ml chloramphenicol were inoculated with ∼60 ml of overnight culture to give an OD_600_ of 0.1. The culture was grown to an OD_600_ of 0.8 and then induced for 6 hrs with 0.5 mM IPTG, at 25°C. The cell pellet was suspended in 20 mls Ni-NTA buffer A (20 mM HEPES-NaOH (pH 7.4), 250 mM NaCl, 10% glycerol) with 1X protease inhibitor cocktail (Roche) and 1 mM β-mercaptoethanol. A micro fluidizer was used to lyse the cells, followed by a 30 minute centrifugation (12,000 rpm, F13 rotor) at 4°C.

### DDK purification

DDK was purified step-wise using Nickel-NTA, SP Fast Flow, and S-200 columns. The cell lysate containing 35 mM imidazole was applied to a 25 ml Ni-NTA column, washed with 20 column volumes, and then eluted with a 250 ml 35 mM-150 mM imidazole gradient. DDK protein fractions (∼115 mM imidazole) were pooled and dialyzed overnight at 4°C against 20 mM HEPES-NaOH, pH 7.4, 1 mM EDTA, 10% glycerol with no imidazole. The dialysate was then passed over three 5 ml SP Fast Flow columns (connected in tandem), washed and eluted with a 100 ml 100 mM-0.5 M NaCl gradient. DDK protein fractions (∼0.2 M) were pooled, MgCl_2_ was added to the pooled protein to chelate EDTA, and incubated with PP2C (6His-GST-Hab1) phosphatase using an equivalent milligram amount to the total protein in the pool, and 1/100 equivalent milligram amount of Ulp1 protease to cleave the His6-Smt3 (Sumo) tag at 16°C overnight. DDK was analyzed on 15% SDS gel to check the extent of dephosphorylation and Sumo cleavage (which was usually greater than 95%). The protein pool was loaded onto a second Ni-NTA column (with no imidazole) and flow through fractions containing DDK were pooled, 1 mM EDTA was added to chelate free Ni^++^, and dialyzed overnight at 4°C against 20 mM HEPES(pH 7.4), 100 mM NaCl, 1 mM EDTA. The protein was concentrated using 30,000 MWCO spin concentrator (Amicon Ultra, Millipore) at 4°C to a final volume of 10 ml. Concentrated protein was loaded onto a 300 ml S-200 gel exclusion column (Amersham-Pharmacia). HsCdc7-Dbf4 eluted at ∼150 kDa, close to the dimer value of 110 kDa. Total yield was typically 6 to 8 mg.

### *In vitro* kinase activation assays

20 ng of purified human DDK was pre-incubated with increasing concentrations of each DDK inhibitor for 5 min. Then 10 µCi (γ)-^32^P ATP and 1.5 µM cold ATP were added in a buffer containing 50 mM Tris-HCl (pH 7.5), 10 mM MgCl_2_, and 1 mM DTT and incubated for 30 min at 30°C. The proteins were denatured in 1X Laemmli buffer at 100°C followed by SDS-PAGE and autoradiography on HyBlot CL film (Denville Scientific, Inc.). Auto-phosphorylation of DDK was used as an indicator of its kinase activity. ^32^P-labeled bands were quantified using ImageJ and the IC_50_ values were calculated using GraphPad (Prism 6).

### Analysis of cell viability

For assays in 96 well plates 2500 cells were plated per well. After 24 hours, cells were treated with small molecule inhibitors and incubated for 72 hours at 37°C. Subsequently the cells were lysed and the ATP content was measured as an indicator of metabolically active cells using the CellTiter-Glo assay (Promega). IC_50_ values were calculated using the GraphPad software. For assays in six well plates, 100,000 cells were plated per well. After 24 hours, cells were treated with small molecule inhibitors and incubated for varying time points. Cells were trypsinized and a suspension was made in 5 ml of phosphate buffered saline. 30 µl of this suspension was mixed with 30 µl of CellTiter-Glo reagent followed by a 10-minute incubation at room temperature. Luminescence was measured using EnVision 2104 Multilabel Reader (PerkinElmer) and BioTek Synergy Neo Microplate Reader.

### Analysis of Caspase 3/7 activity

5,000 cells per well were plated in a 96 well plate. After 24 hours, cells were treated with small molecule inhibitors and incubated for 24 hours at 37°C. Caspase 3/7 activity and viable cell number were then measured using the Caspase-Glo 3/7 assay (Promega) and CellTiter-Glo assay (Promega), respectively. The “Caspase activity per cell” was obtained by normalizing total Caspase activity to cell number.

### Immunoblot Analysis

Whole cell extracts were prepared by re-suspending the pellets in RIPA buffer (150 mM NaCl, 1% NP-40, 0.5% sodium deoxycholate, 0.1% SDS, 50 mM Tris HCl, pH 8) containing protease inhibitors (100 µM PMSF, 1 mM Benzamide, 2.5 µg/ml Pepstatin A, 10 µg/ml Leupeptin, and 10 µg/ml Aprotinin) and phosphatase inhibitors (1 mM each NaF, Na_3_VO_4_ and Na_4_P_2_O_7_). Protein concentration was measured using the BCA protein assay kit (Pierce) according to manufacturer's protocol. Equal amounts of protein were subjected to SDS-PAGE and transferred to a nitrocellulose membrane (Millipore). Transfer efficiency and equal loading was confirmed by Ponceau S staining. Following primary and secondary antibody treatments, proteins were visualized using SuperSignal West Pico solutions (Thermo Scientific). Anti-Mcm2 and anti-S53-phospho-Mcm2 antibodies were purchased from Bethyl Laboratories; anti-β-actin was from Sigma; anti-mouse and anti-rabbit HRP antibodies were from GE Healthcare; and anti-Cdc7 and anti-Dbf4 antibodies were described previously [26].

### Thermal Stability Shift Assay (TSA)

All reactions were incubated in a 10 µl final volume and assayed in 96-well plates using 20 x SYPRO Orange (Invitrogen) and 200 µg/ml purified DDK [50]. Reactions were incubated with inhibitor compounds on ice for 30 minutes. Compounds from four kinase inhibitor libraries (Calbiochem I, II, III, Tocriscreen Inhibitor Toolbox) were screened at 20 µM for T_m_ increases with a total DMSO concentration of 2% or less. Thermal melting experiments were carried out using the StepOnePlus Real-Time PCR System (Applied Biosystems) melt curve program with a ramp rate of 1°C and temperature range of 15°C to 85°C. Subsequent TSAs on the 12 hits obtained were carried out as above but in triplicate and using a 200-fold range of inhibitor concentrations. Data analysis was performed as described [50]. Melting temperatures (T_m_) were calculated by fitting the sigmoidal melt curve to the Boltzmann equation using GraphPad Prism, with R^2^ values of >0.99. The difference in T_m_ values calculated for reactions with and without compounds is ΔT_m_.

## Results

### DDK inhibitors exhibit very different cellular potencies

**Figure 2:**
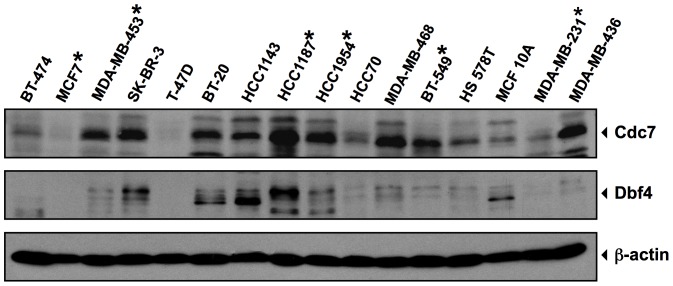
DDK is overexpressed in multiple breast cancer cell lines. Immunoblots showing the expression levels of Cdc7 and Dbf4 in tumor cell lines. β-actin levels indicate equal loading of proteins.

**Figure 3:**
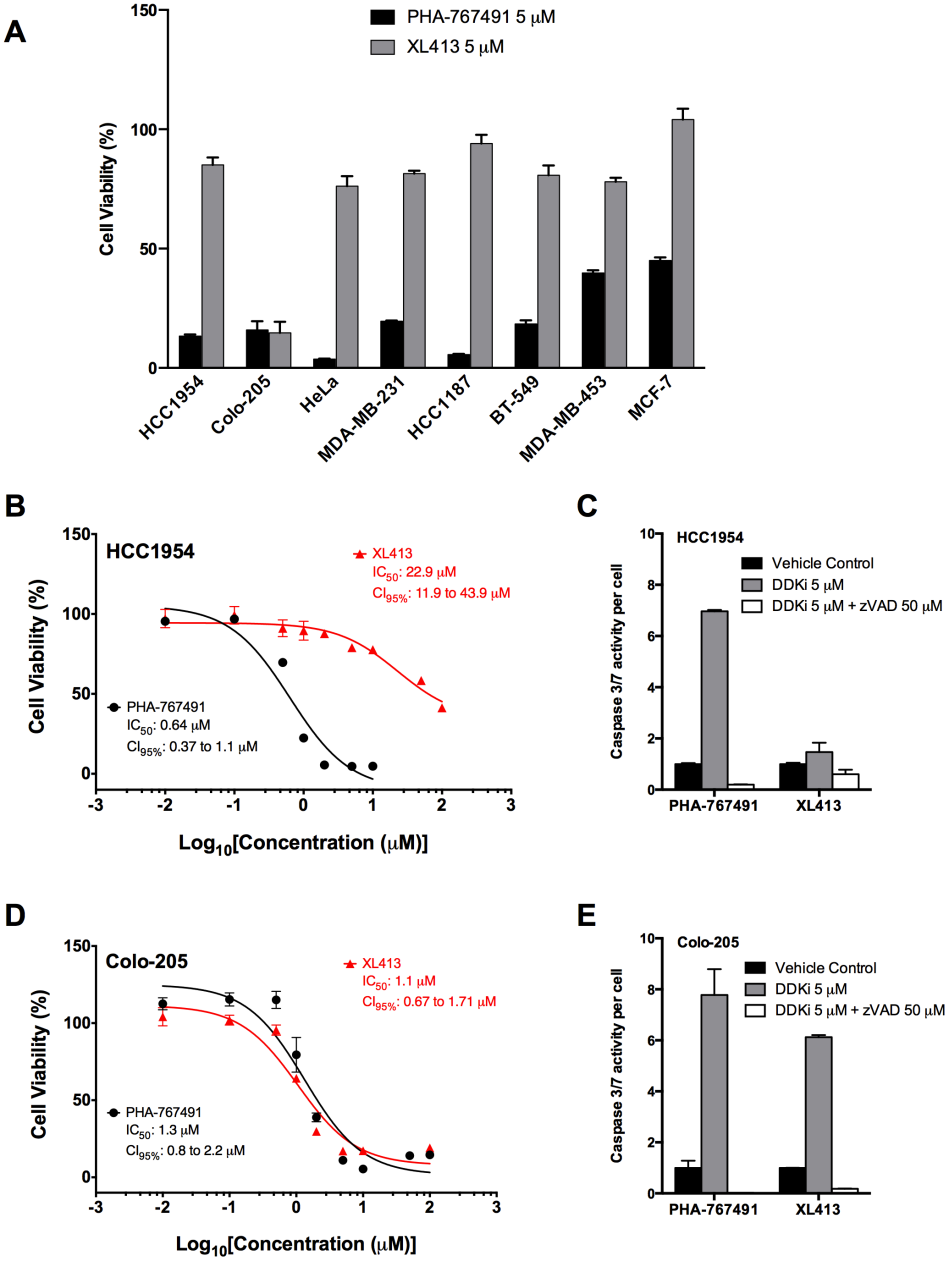
Two DDK inhibitors, PHA-767491 and XL413, exhibit differential activity against cultured tumor cells. (**A**) Eight tumor cell lines were treated with 5 µM of each DDK inhibitor and cell viability was measured 72 hrs post drug addition. To determine the IC_50_ values, HCC1954 cells were treated with increasing concentrations of PHA-767491 or XL413 (**B**) and the cell viability was measured 72 hrs post drug addition. Colo-205 cells were treated with increasing concentrations of PHA-767491 or XL413 (**D**) and the cell viability was measured 72 hrs post drug addition. The extent of apoptosis induced by the compounds in each cell line relative to vehicle control was measured by Caspase 3/7 activity and is indicated in (**C, E**). All data represent the mean of at least three separate measurements +/− SD and were highly reproducible on separate days.

We screened a panel of 15 breast cancer cell lines for Cdc7 and Dbf4 expression using monoclonal antibodies against each subunit [26]. The majority of these express the DDK subunits equivalent to or higher than MCF10A, an immortalized but non-tumorigenic mammary epithelial cell line that served as a non-tumor control (Figure 2). We used PHA-767491 and XL413 to inhibit DDK in a panel of six breast cancer cell lines that overexpress DDK at various levels (marked with asterisks in Figure 2). Both compounds have been reported to have anti-proliferative activities in the low micromolar range [43], [49]. As controls, we compared these results to PHA-767491 treatment of HeLa cells and XL413 treatment of Colo-205 cells, which inhibit DDK and induce cell death. Since Cdc7 kinase is an essential protein, inhibiting its activity should significantly slow or arrest cell proliferation. PHA-767491 significantly inhibited proliferation in all cell lines tested (Figure 3A, values are plotted relative to vehicle controls). PHA-767491 was most effective on the HeLa and HCC1187 cell lines and had the least effect on the MCF-7 [43] and the MDA-MB-453 cell lines: 2-fold and 2.5-fold inhibited, respectively. In contrast, XL413 was anti-proliferative only in the Colo-205 cells (Figure 3A).

We then examined the potency profiles of both compounds in more detail using the XL413-sensitive (Colo-205) and XL413-resistant (HCC1954) cell lines. Cells were incubated in presence of increasing concentrations of the inhibitors for 72 hours at 37°C followed by cell viability measurements. PHA-767491 inhibited proliferation in both cell lines with an IC_50_ of 0.64 µM in HCC1954 cells and 1.3 µM in Colo-205 cells (Figure 3, B and D), consistent with the average 3.17 µM IC_50_ value calculated using a panel of 61 tumor cell lines [43]. In contrast, XL413 had an IC_50_ of 22.9 µM in HCC1954 cells and 1.1 µM in Colo-205 cells (Figure 3, B and D). In correspondence with the viability data, PHA-767491 induced apoptosis in both the HCC1954 and Colo-205 cells, but XL413 induced apoptosis only in the Colo-205 cells (Figure 2, C and E). XL413 was not a specific inhibitor of colorectal tumor lines because it had limited effects on two additional colorectal tumor cell lines: XL413 had 40- to 60-fold higher IC_50_ values than PHA-767491 on these lines (Figure S1 in File S1).

### PHA-767491 and Xl413 are potent DDK inhibitors *in vitro*

**Figure 4:**
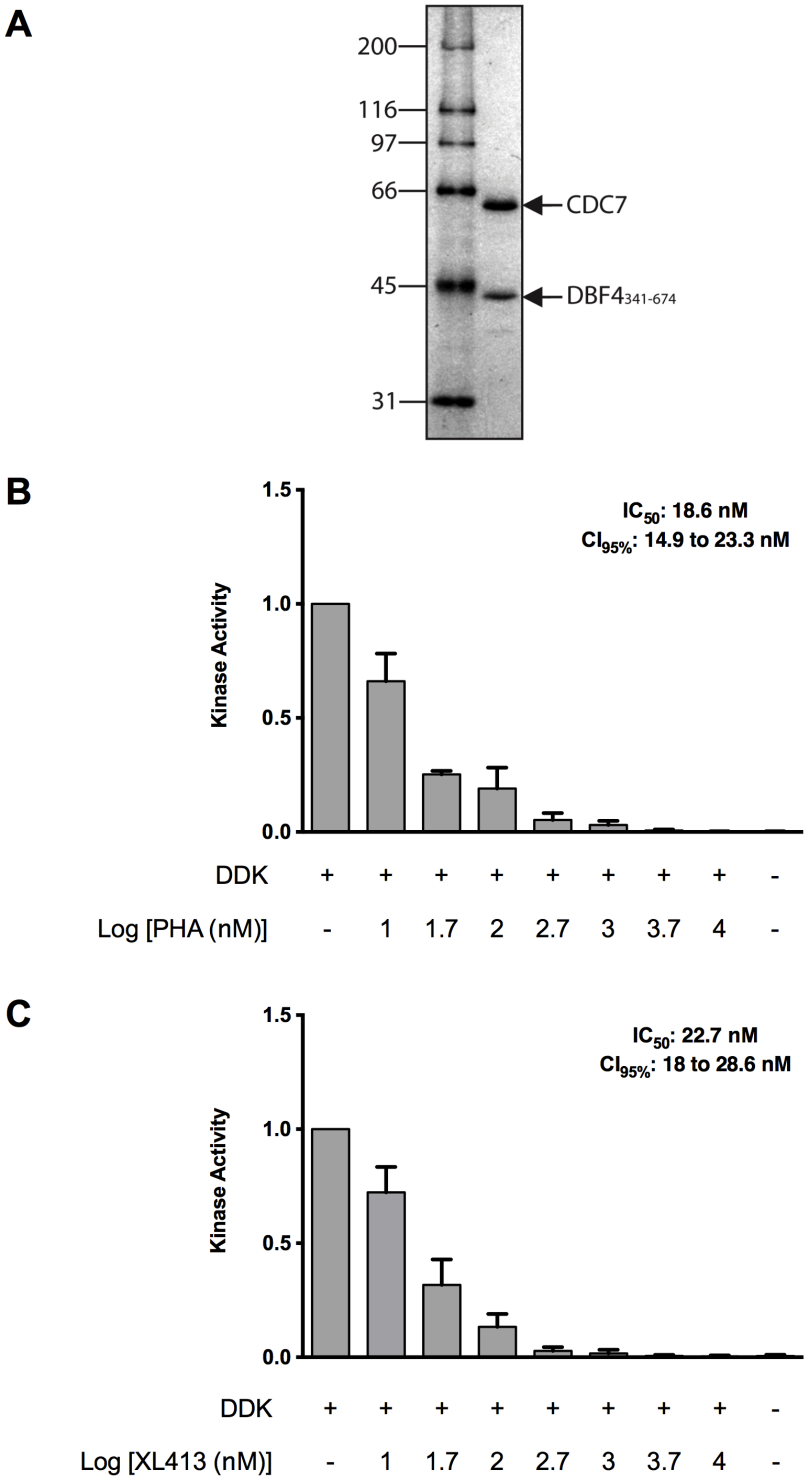
PHA-767491 and XL413 are similarly effective DDK inhibitors *in vitro*. (**A**) Coommassie-stained gel showing 1 µg purified DDK from bacterial cells. (γ)-^32^P ATP DDK kinase assays in presence of increasing concentrations of PHA-767491 (**B**) or XL413 (**C**). Kinase activities represent the mean of four independent measurements +/− SD on separate days.

The poor potency of XL413 on most tumor cell lines could be because the synthesized compound is not an effective kinase inhibitor. To test this possibility, we purified recombinant DDK and then measured the IC_50_ values of both XL413 and PHA-767491 on purified kinase. We co-expressed His6-SUMO-Cdc7 and Dbf4 in bacterial cells and then purified the complex as described in Materials and Methods. Briefly, DDK was bound to a Ni-NTA column followed by elution and removal of the His6-SUMO tag. Untagged DDK was then fractionated over an SP Fast Flow column followed by separation on an S-200 gel filtration column. Kinase assays were performed with purified DDK (Figure 4A) in the presence of increasing concentrations of each inhibitor (Figure 4, B and C). Both PHA-767491 and XL413 were effective DDK inhibitors *in vitro* as shown previously [16], [42], [49] with IC_50_ values of 18.6 nM and 22.7 nM, respectively. Since both compounds are effective DDK inhibitors, the relative cell viability profiles indicate that XL413 is deficient in acting on its target inside the cell.

### XL413 is defective in inhibiting DDK-dependent Mcm2 phosphorylation in HCC1954 cells

Effective cellular uptake of the DDK inhibitor should compromise DDK activity *in vivo*. Among the many targets of DDK are components of the replicative Mcm2–7 helicase. Serine 53 of Mcm2 subunit is a well-characterized target site for DDK mediated phosphorylation [24]. We quantitated levels of phosphorylation on this site as a measure of DDK activity *in vivo*. HCC1954 cells were incubated in presence of 1 µM PHA-767491, 2 µM PHA-767491 or 5 µM XL413. Cells were then harvested at 0, 24, 48, and 72 hours post drug addition to measure viable cells and Mcm2 phosphorylation by immunoblotting.

**Figure 5:**
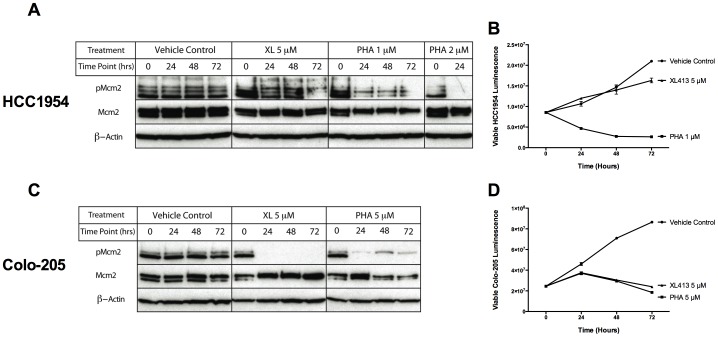
XL413 is defective in inhibiting DDK-dependent Mcm2 phosphorylation in HCC1954 cells but is effective in Colo-205 cells. (**A**) Immunoblots showing Mcm2 phosphorylation in HCC1954 cells or (**C**) Colo-205 cells in the presence of DMSO, PHA-767491, or XL413. (**B**) Cell proliferation profile of HCC1954 cells or (**D**) Colo-205 cells in presence of DMSO, PHA-767491, or XL413. The cell viability data represent the mean of at least two measurements +/− SD and were highly reproducible on different days.

2 µM PHA-767491 completely abolished Mcm2 phosphorylation by 24 hours in HCC1954 cells (Figure 5A), corresponding with its affect on cell growth and viability (Figure 5B). In the same cell line, 1 µM PHA-767491 resulted in very little residual Mcm2 phosphorylation from 24 to 72 hours and was also effective in inhibiting cell viability and inducing cell death. In contrast, XL413 did not inhibit Mcm2 phosphorylation at 24 hrs, even at a higher concentration of 5 µM (Figure 5A) and there was only a modest decrease in Mcm2 phosphorylation at 72 hours. This effect was also seen in the cell viability assay, where XL413 treated cells grew only slightly poorer than the vehicle treated cells (Figure 5B).

Since both compounds were effective inhibitors in the Colo-205 cells, we examined Mcm2 phosphorylation in these cells following drug addition. Again, 5 µM PHA-767491 completely abolished Mcm2 phosphorylation by 24 hours and was very effective in inducing cell death (Figure 5, C and D). However, unlike in HCC1954 cells, XL413 was a very effective inhibitor of DDK activity in Colo-205 cells. 5 µM of XL413 completely abolished Mcm2 phosphorylation at 24 hrs and was also as effective as PHA-767491 in inducing cell death (Figure 5, C and D). These results show that the two DDK inhibitors exhibit very different profiles in cell lines despite the fact that both compounds are highly effective kinase inhibitors *in vitro*. Our data suggest that XL413 is not taken up effectively into many cell lines or is metabolized quickly or modified to an inactive form.

### Screen to determine cross reactivity of known kinase inhibitors with DDK

To identify additional chemical structures that are capable of inhibiting DDK, we tested a panel of ∼400 kinase inhibitors against purified DDK in a thermal stability shift assay (TSA) [50]. In this assay, inhibitor compounds were incubated with purified DDK and then screened with an increasing temperature gradient to determine the point at which they denature (relative to DDK alone) by following fluorescence changes of the dye SYPRO Orange, which binds to hydrophobic surfaces on unfolded proteins. Inhibitor compounds that bind within the DDK ATP binding pocket are predicted to stabilize the kinase, and ΔT_m_ values (see Materials and Methods) of 2°C or greater are considered significant hits. Experimental results of the 400 compound screen are listed in Figure S2 in File S1. We identified 12 compounds that caused significant temperature shifts: 11 compounds increased the T_m_, and 1 compound (Genistein) decreased the T_m_ (Table S1 in File S1).

**Figure 6:**
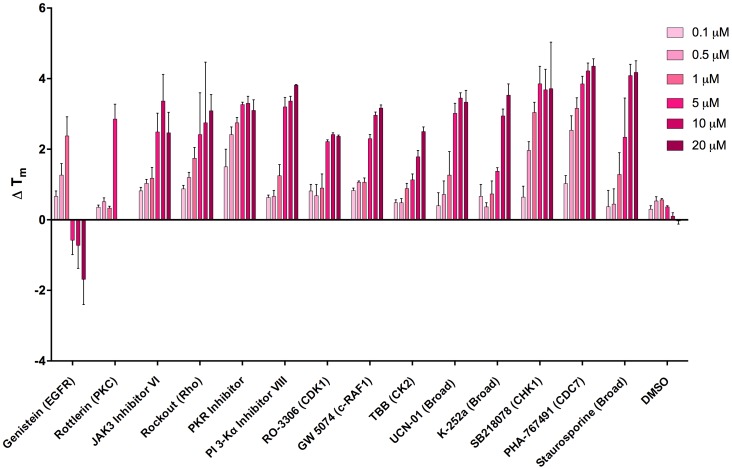
DDK thermal stability shift assays (TSA) using known kinase inhibitors. Increasing concentrations of 12 hit compounds discovered in a 400 compound screen (Table S1 in File S1) were screened against purified DDK using the TSA. PHA-767491 (DDK specific inhibitor), staurosporine (broad spectrum kinase inhibitor) and DMSO are shown as controls. The data represent the mean of triplicate measurements +/− SEM.

To estimate the affinity of each compound for DDK we measured ΔT_m_ values for these 12 compounds across a 200-fold range of inhibitor concentrations and compared these values to PHA-767491 (a specific DDK inhibitor), staurosporine (a broad spectrum protein kinase inhibitor), and DMSO as a vehicle control. The data shown in Figure 6 represent an average of three independent measurements. The compound genistein, which is an EGFR inhibitor, was unusual in that it increased ΔT_m_ at lower inhibitor concentrations and then decreased ΔT_m_ at 5, 10 and 20 µM concentrations. The initial screen was carried out with 20 µM inhibitor and explains why genistein was scored as decreasing the T_m_. Perhaps this compound binds to the DDK ATP binding pocket but at higher concentrations disrupts Cdc7-Dbf4 binding. Each of the other 11 compounds has positive ΔT_m_s. Examination of the compound titrations reveals that three inhibitors had comparable profiles to PHA-767491 in that they induced a ΔT_m_ of ∼2 or more beginning at a 1 µM concentration: a Rho kinase inhibitor (Rockout), a protein kinase R (PKR) inhibitor, and a Chk1 kinase inhibitor (SB218078). Four additional compounds, the JAK3 inhibitor VI, PI3-Kα inhibitor VIII, UCN-01, and K-252a gave a 3-fold or higher ΔT_m_ at 5, 10 and 20 µM concentrations.

**Figure 7:**
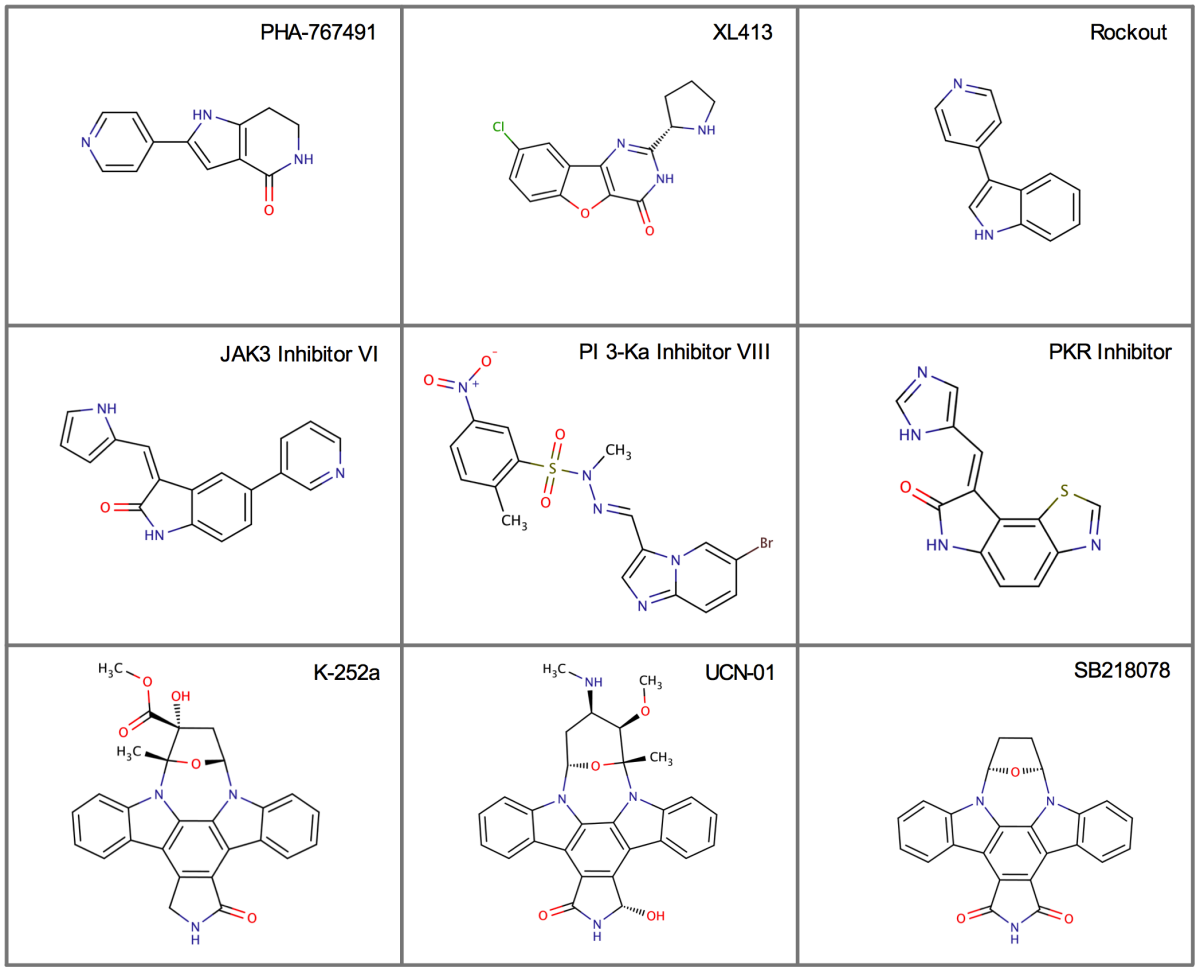
Structures of potential DDK inhibitors. The structures of the 7 top compounds from the TSA inhibitor titrations are shown together with PHA-767491 and XL413 structures for comparison (see text and Table S1 in File S1 for further details). Three compounds are derived from staurosporine (bottom row) but the remaining four compounds fall into distinct structural classes.

**Figure 8:**
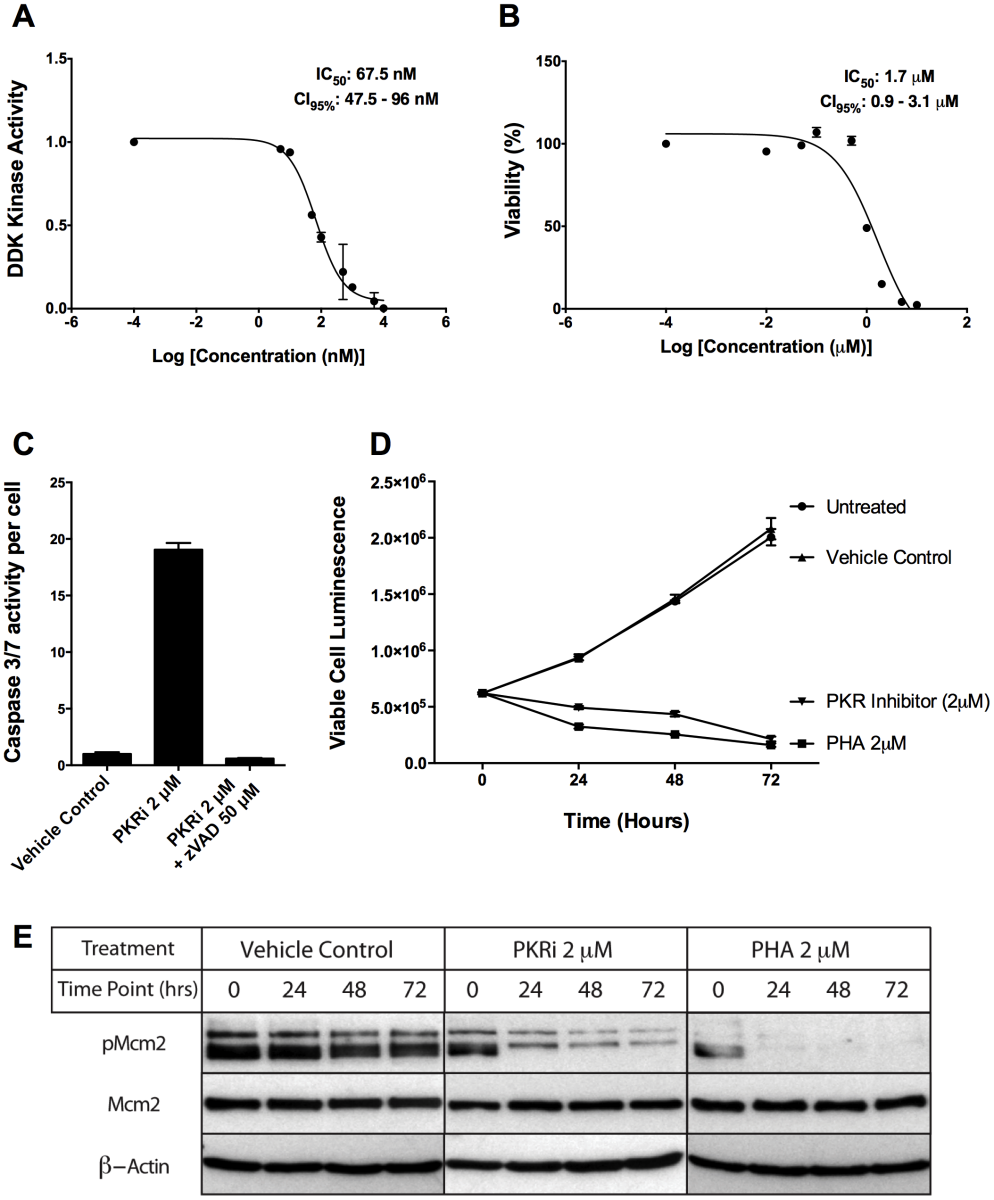
A PKR inhibitor also inhibits DDK activity and induces apoptosis in breast cancer cells. IC_50_ values for the PKR inhibitor (shown in Figure 7) were determined against purified DDK (**A**) and HCC1954 cells (**B**). (**C**) Caspase 3/7 assays showing that apoptosis was strongly induced at 24 hours following PKR inhibitor addition and this was eliminated using the pan-caspase inhibitor z-VAD. The PKR inhibitor causes a similar decrease in viability on HCC1954 cells to PHA-767491 over time (**D**) and also inhibits Mcm2 phosphorylation in cells, a known DDK target (**E**). The measurements in panels A–D represent the averages of at least two measurements +/− SD and were highly reproducible.

The structures of the top compounds in the TSA screen are shown in Figure 7, revealing a broad range of structural classes. K-252a is naturally occurring alkaloid related to staurosporine that inhibits a broad variety of protein kinases including serine/threonine kinases and tyrosine kinases of the Trk family [51], [52]. So, inclusion of K-252a in this list (like staurosporine) is perhaps not surprising. Since it is very likely that the inhibitors we recovered in the TSA screen stabilize DDK by their ability to bind in the ATP binding pocket and inhibit DDK, we performed kinase assays using the top six compounds. Kinase assays revealed that they are indeed DDK inhibitors (Figure S3 in File S1). The Chk1 (SB218078) and the PKR inhibitors were the best compounds *in vitro* and inhibited DDK with IC_50_s of 19.3 nM and 67.5 nM, respectively (Figure 8A and Figure S3 in File S1). Interestingly, the ΔT_m_ profiles of the Chk1 and PKR inhibitors look strikingly like PHA-767491, raising the possibility that these compounds inhibit DDK in cells. Although SB218078 is derived from staurosporine, it is a potent inhibitor of Chk1 [53]. The structures of the other top hits, the PKR inhibitor and Rockout, are not derived from staurosporine and also differ from known DDK inhibitors (Figure 1).

We tested whether the PKR and Chk1 inhibitors would alter cell growth and inhibit Mcm2 phosphorylation in the HCC1954 breast cancer cell line, which would be strong evidence that they inhibit DDK in cells. Increasing amounts of the PKR inhibitor were incubated with HCC1954 cells over 72 hours, which resulted in a large decrease in the number of viable cells relative to vehicle control (Figure 8B, IC_50_ of 1.7 µM). The large decrease in cell viability was likely the result of significant apoptosis because 2 µM PKR inhibitor increased Caspase 3/7 activity 20-fold relative to the DMSO control and this was blocked by the pan-caspase inhibitor z-VAD (Figure 8C). 2 µM PKR inhibitor impaired cell growth over a 72 hour time course similar to 2 µM PHA-767491 (Figure 8D) and also strongly inhibited Mcm2 phosphorylation in the HCC1954 cells (Figure 8E). These results strongly suggest that the PKR inhibitor is blocking DDK activity in this cell line. We saw the same trend with the Chk1 inhibitor, although it had a reduced ability to block cell growth, induce apoptosis, and inhibit Mcm2 phosphorylation relative to the PKR and PHA-767491 inhibitors (Figure S4 in File S1).

## Discussion

Small molecule inhibitors have been successfully employed both in the clinic and laboratory. Despite being initially regarded as too non-specific for deployment in therapy, small molecule kinase inhibitors have emerged as frontrunners in drug development, especially against cancer [54]. Clinically useful molecules are often called ‘drugs’ while the ones used for studying protein functions in the laboratory are called ‘chemical probes’ [55], [56]. Both the groups share a basic requirement of high potency against the target of interest. While drugs need to act effectively against the targeted disease and exhibit good pharmacokinetic properties in a physiological setting [56], for chemical probes target specificity is of paramount importance [55]. Small molecule inhibitors of DDK are attractive both as drugs as well as chemical probes.

Since the initial description of the tumor specific cell killing observed in response to depletion of DDK, several DDK inhibitors have been synthesized. Very different families of chemical moieties have been shown to exhibit DDK inhibitory activities. Nerviano Medical Sciences, Roche, Abbot, Exelixis, and Amgen have developed and characterized DDK inhibitors. Although DDK inhibitors may be effective anti-cancer drugs, these molecules are also very important for understanding the roles of this multifunctional kinase. As probes, DDK inhibitors would complement the traditional RNAi techniques, which can also have off-target effects [57]. RNAi mediated silencing leads to a gradual loss of protein whereas an inhibitor impacts kinase activity and not necessarily protein abundance [57]. Chemical inhibitors could also be important in studying the non-kinase roles of DDK.

Two inhibitors of DDK have received more characterization than others: the first was the prototype DDK inhibitor PHA-76749 followed by the highly selective benzofuropyrimidinone XL413. An X-ray crystal structure of DDK in association with both DDK inhibitors has been solved recently [16]. The tighter binding of XL413 in the binding pocket of DDK along with its more extensive associations with the non-conserved residues of the active site is thought to be the reason for the superior selectivity profile of XL413. The cellular potency data provided with the initial characterization of XL413 [49] along with the crystal structure evidence made it the best in class DDK inhibitor. XL413 seemed an ideal chemical probe for studies of DDK function in normal and in tumor cells.

It was therefore surprising that in most cell lines we tested XL413 fared very poorly when compared to PHA-767491. This led us to perform a comparative analysis of the biochemical characteristics of both inhibitors. Both inhibitors were quite effective in inhibiting purified DDK complex *in vitro*. Although the cancer cell lines had varying levels of DDK, they all responded well to PHA-767491. XL413, however, had almost no effect on nine of the ten cell lines in our panel. It also did not induce cell cycle arrest in majority of cell lines, indicating that DDK activity was not being inhibited *in vivo*. This was corroborated by the Mcm2 phosphorylation analysis in XL413-sensitive Colo-205 cells and XL413-resistant HCC1954 cells. The majority of the original cellular potency profile for XL413 was provided with one cell line, Colo-205. In our analysis, Colo-205 was the sole cell line highly responsive to XL413. Taken together, our analyses suggest that XL413, while exhibiting impressive chemical characteristics and selectivity, is a poor chemical probe for cell lines.

As described by Workman and Collins [55], the effectiveness of an inhibitor as a chemical probe is dependent on its (1) chemical properties (2) biological potency (3) biological selectivity and (4) its context of use. Since XL413 is a product of a high throughput drug-screening program and must have satisfied multiple criteria for selection as lead compound, it is expected to exhibit good pharmacokinetic properties. XL413 was shown to be a highly potent inhibitor with IC_50_ values in single digit nanomolar range. Moreover, it was highly selective for DDK over a panel of 100 kinases. With such properties, the poor growth suppressive properties of XL413 among so many cell lines cannot be easily explained. We also performed analyses with XL413 purchased from a separate commercial supplier, MedKoo Inc, however this compound had identical cellular potency profiles as the compound synthesized by CGeneTech (Figure S5 in File S1). Both HCC1954 and Colo-205 cells were cultured in RPMI-1640 media supplemented with 10% fetal bovine serum. Since, the inhibitor functions well in Colo-205 cells, precipitation of XL413 in media cannot be the reason for its inactivity. Possible reasons for its compromised activity on cell lines include poor permeability through the cell membrane, degradation by metabolic enzymes, modification to an inactive form, or higher sensitivity to efflux transporters. In principle, these possible deficits could be identified and then circumvented through synthesis of additional chemical derivatives.

Our analysis of XL413 highlights a need for additional biologically active DDK inhibitors. Most ATP competitive inhibitors were optimized by structure activity relationship (SAR) studies on existing scaffolds of chemical inhibitors. PHA-767491 and XL413 were optimized from scaffolds for MK2 and PIM inhibitors, respectively [58], [59]. To identify further chemical scaffolds for development of DDK inhibitors, we tested if any well-known kinase inhibitors cross-reacted with human DDK. This is a possibility since ATP-competitive kinase inhibitors bind within a related ATP-binding pocket. Using a TSA screen, we identified 12 small molecules that significantly shifted the thermal stability of DDK. Several of these functioned comparatively to PHA-767491 in the assay: the Rho kinase inhibitor (Rockout), the PKR inhibitor, and the Chk1 inhibitor (SB218078). These compounds fall into different structural classes (Figure 7) indicating that significant chemical space is available for new DDK inhibitor development. Interestingly, UCN-01, also a Chk1 inhibitor related to staurosporine [60], was also identified in our screen and showed a high affinity for DDK. This raises the possibility that more potent and selective derivatives of staurosporine might be designed against DDK. It also raises the possibility that reported biological effects due to Chk1 inhibition may be enhanced by the ability of SB218078 and/or UCN-01 to also inhibit DDK. Rockout is a pyridine-substituted indole derivative and so is somewhat related to PHA-767491. However, the position of the pyridine moiety on the indole ring of Rockout is quite different from the geometry of PHA-767491. The PKR inhibitor also falls into a distinct structural class from either PHA-767491 or XL413.

It was noteworthy that the PKR inhibitor blocked the growth of HCC1954 breast cancer cells, induced apoptosis, and inhibited DDK-mediated Mcm2 phosphorylation nearly as well as the lead DDK inhibitor PHA-767491. RNA-dependent protein kinase (or PKR) is ubiquitously expressed protein that blocks protein synthesis in response to a number of stresses and impacts both neurodegenerative diseases and cancer through its ability to promote apoptosis [61]. The particular PKR inhibitor we used inhibited PKR with an IC_50_ of 210 nM [62] but inhibited DDK with an IC_50_ of ∼70 nM *in vitro* (Figure 8A), and so should be classified as a dual PKR/DDK inhibitor. Whether the PKR inhibitor induced apoptosis in HCC1954 cells due to inhibiting DDK activity, PKR activity or both remains to be determined. In summary, our results highlight the cross-reactivity of several kinase inhibitors with DDK and also reveal an opportunity to develop more potent, biologically active DDK inhibitors for future evaluation.

## Supporting Information

File S1 **Supporting files.** **Figure S1**, Two DDK inhibitors, PHA-767491 and XL413, exhibit differential activity against cultured HCT116 colon cancer cells. To determine the IC_50_, HCT116 (p53^+/+^) cells were treated with increasing concentrations of PHA-767491 or XL413 (**A**) and the cell viability was measured 72 hrs post drug addition. HCT116 (p53^-/-^) cells were treated with increasing concentrations of PHA-767491 or XL413 (**B**) and the cell viability was measured 72 hrs post drug addition. All data represent the mean of at least three separate measurements +/− SD. **Figure S2**, DDK thermal stability shift assays (TSA) screen using 400 known kinase inhibitors. **Figure S3**, Top hits identified by TSA screen inhibit DDK *in vitro*. (γ)-^32^P ATP DDK kinase assays in presence of increasing concentrations of SB 218078 (**A**), PKR Inhibitor (**B**), UCN-01 (**C**), JAK3 Inhibitor VI (**D**), Rho Kinase Inhibitor III (**E**), and PI3-Kα Inhibitor VIII (**F**). Kinase activities represent the mean of two independent measurements +/− SD on separate days. **Figure S4**, The Chk1 inhibitor (SB 218078) inhibits DDK activity and induces low levels of apoptosis in breast cancer cells. IC_50_ values for SB 218078 (structure shown in Figure 7) were determined against purified DDK (**A**) and HCC1954 cells (**B**). Caspase 3/7 assays show that low levels of apoptosis were induced at 24 hours following SB 218078 addition and this was eliminated using the pan-caspase inhibitor z-VAD (**C**). SB 218078 induces cell growth arrest in HCC1954 cells over time (**D**) and also modestly inhibits Mcm2 phosphorylation in cells, a known DDK target (**E**). The measurements in panels A-D represent the averages of at least two measurements +/− SD and were highly reproducible. **Figure S5**, XL413 acquired from the commercial supplier (MedKoo Biosciences, North Carolina) behaved similar to the chemically synthesized compound. To determine the IC_50_ values, HCC1954 and Colo-205 cells were treated with increasing concentrations of XL413 (MedKoo) and the cell viability was measured 72 hrs post drug addition. All data represent the mean of at least three separate measurements +/− SD and were highly reproducible on separate days. **Table S1**, Characteristics of top kinase inhibitors discovered in TSA screen.
(PDF)
