# Supplementary material for: The Potent Cdc7-Dbf4 (DDK) Kinase Inhibitor XL413 Has Limited Activity in Many Cancer Cell Lines and Discovery of Potential New DDK Inhibitor Scaffolds
Source: PLoS One. 2014 Nov 20;9(11):e113300. doi: 10.1371/journal.pone.0113300 (PMC4239038; doi:10.1371/journal.pone.0113300)
Supplement: File S1 — Supporting files. Figure S1, Two DDK inhibitors, PHA-767491 and XL413, exhibit differential activity against cultured HCT116 colon cancer cells. To determine the IC50, HCT116 (p53+/+) cells were treated with increasing concentrations of PHA-767491 or XL413 (A) and the cell viability was measured 72 hrs post drug addition. HCT116 (p53-/-) cells were treated with increasing concentrations of PHA-767491 or XL413 (B) and the cell viability was measured 72 hrs post drug addition. All data represent the mean of at least three separate measurements +/− SD. Figure S2, DDK thermal stability shift assays (TSA) screen using 400 known kinase inhibitors. Figure S3, Top hits identified by TSA screen inhibit DDK in vitro. (γ)-32P ATP DDK kinase assays in presence of increasing concentrations of SB 218078 (A), PKR Inhibitor (B), UCN-01 (C), JAK3 Inhibitor VI (D), Rho Kinase Inhibitor III (E), and PI3-Kα Inhibitor VIII (F). Kinase activities represent the mean of two independent measurements +/− SD on separate days. Figure S4, The Chk1 inhibitor (SB 218078) inhibits DDK activity and induces low levels of apoptosis in breast cancer cells. IC50 values for SB 218078 (structure shown in Figure 7) were determined against purified DDK (A) and HCC1954 cells (B). Caspase 3/7 assays show that low levels of apoptosis were induced at 24 hours following SB 218078 addition and this was eliminated using the pan-caspase inhibitor z-VAD (C). SB 218078 induces cell growth arrest in HCC1954 cells over time (D) and also modestly inhibits Mcm2 phosphorylation in cells, a known DDK target (E). The measurements in panels A-D represent the averages of at least two measurements +/− SD and were highly reproducible. Figure S5, XL413 acquired from the commercial supplier (MedKoo Biosciences, North Carolina) behaved similar to the chemically synthesized compound. To determine the IC50 values, HCC1954 and Colo-205 cells were treated with increasing concentrations of XL413 (MedKoo) and the cell viability was mea [file pone.0113300.s001.pdf]

A

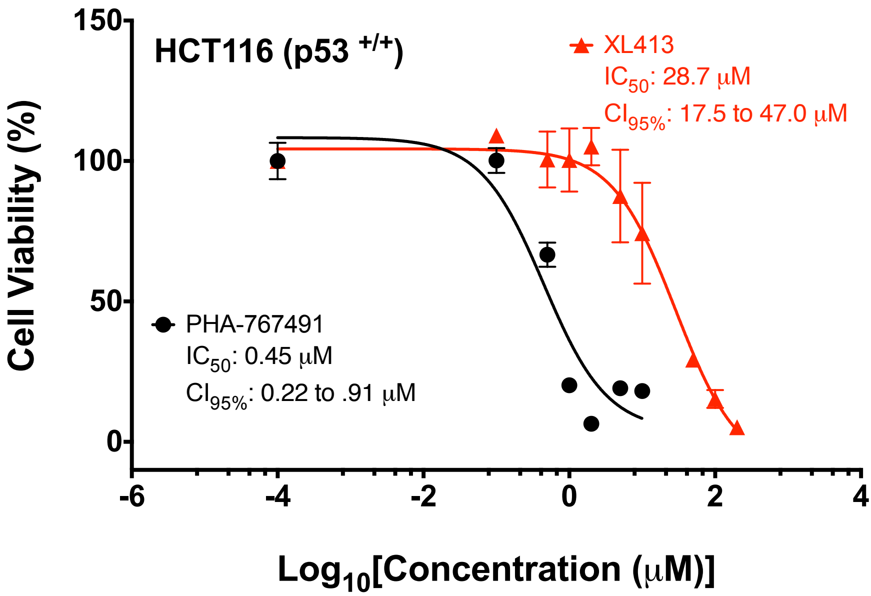
HCT116 (p53 +/+)
XL413
IC50: 28.7 μM
CI95%: 17.5 to 47.0 μM
PHA-767491
IC50: 0.45 μM
CI95%: 0.22 to .91 μM
Cell Viability (%)
150
100
50
0
-6
-4
-2
0
2
4
Log10[Concentration (μM)]

B

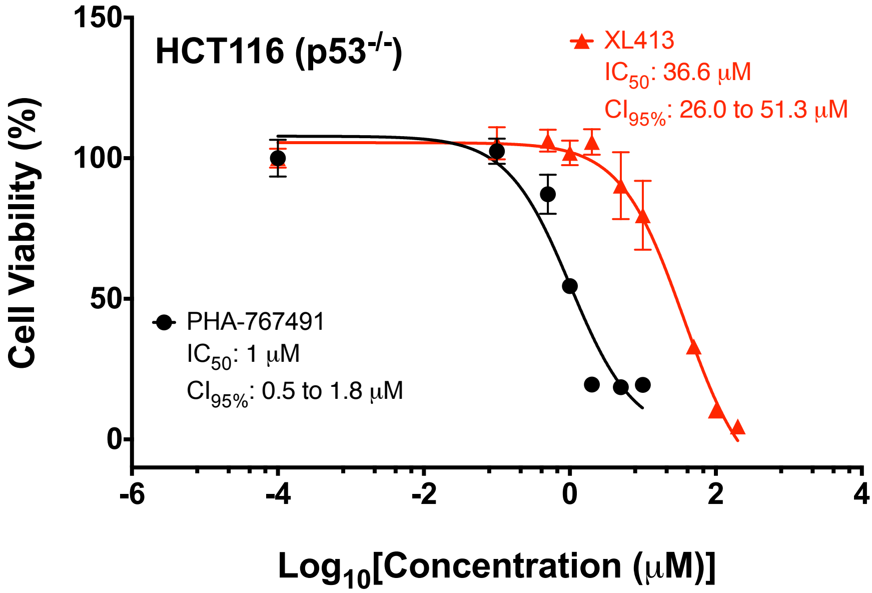
HCT116 (p53-/-)
XL413
IC50: 36.6 μM
CI95%: 26.0 to 51.3 μM
PHA-767491
IC50: 1 μM
CI95%: 0.5 to 1.8 μM
Cell Viability (%)
150
100
50
0
-6
-4
-2
0
2
4
Log10[Concentration (μM)]

Figure S1

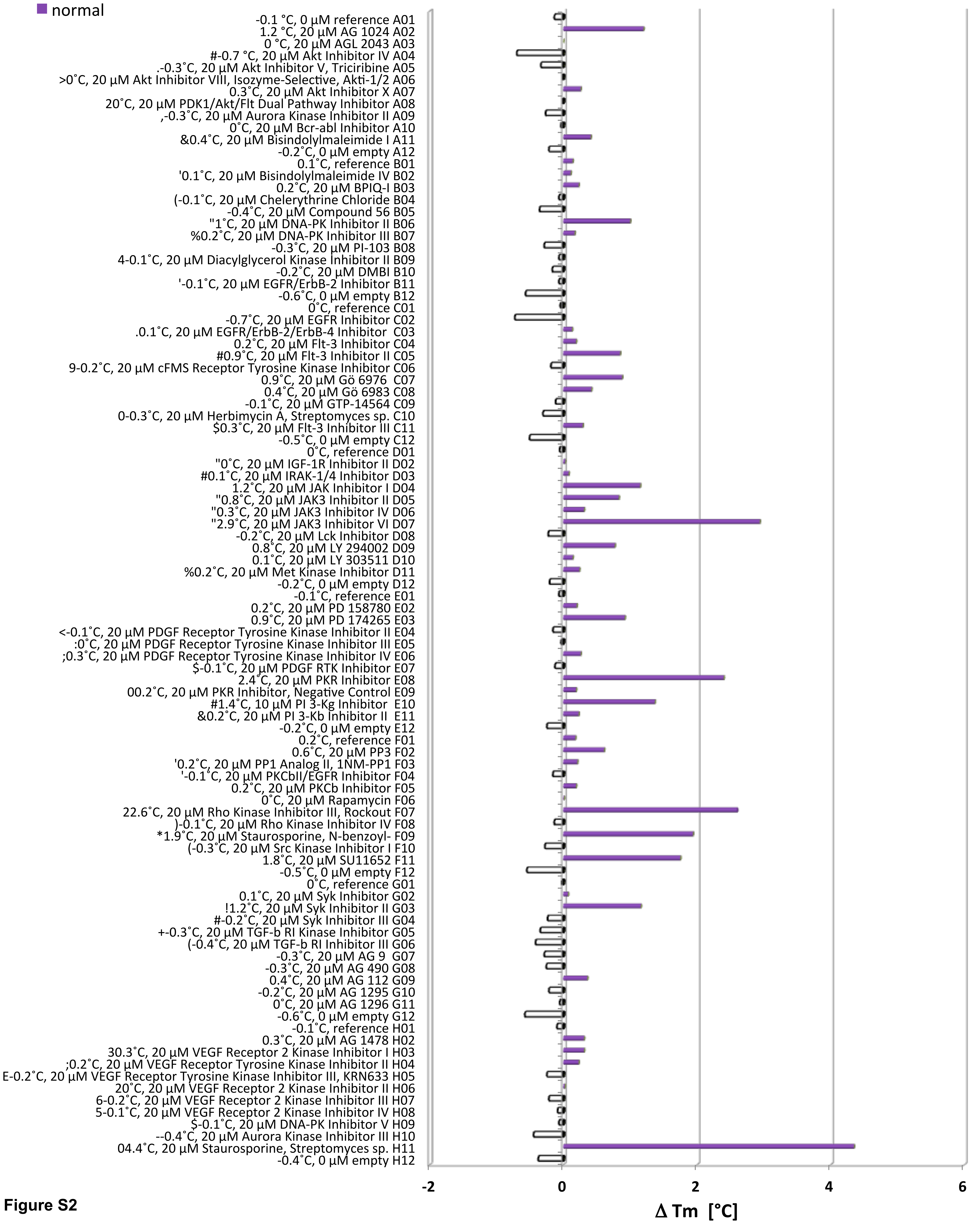
normal
-0.1 °C, 0 µM reference A01
1.2 °C, 20 µM AG 1024 A02
0 °C, 20 µM AGL 2043 A03
#-0.7 °C, 20 µM Akt Inhibitor IV A04
.-0.3˚C, 20 µM Akt Inhibitor V, Triciribine A05
>0˚C, 20 µM Akt Inhibitor VIII, Isozyme-Selective, Akti-1/2 A06
0.3˚C, 20 µM Akt Inhibitor X A07
20˚C, 20 µM PDK1/Akt/Flt Dual Pathway Inhibitor A08
,-0.3˚C, 20 µM Aurora Kinase Inhibitor II A09
0˚C, 20 µM Bcr-abl Inhibitor A10
&0.4˚C, 20 µM Bisindolylmaleimide I A11
-0.2˚C, 0 µM empty A12
0.1˚C, reference B01
'0.1˚C, 20 µM Bisindolylmaleimide IV B02
0.2˚C, 20 µM BPIQ-I B03
(-0.1˚C, 20 µM Chelerythrine Chloride B04
-0.4˚C, 20 µM Compound 56 B05
"1˚C, 20 µM DNA-PK Inhibitor II B06
%0.2˚C, 20 µM DNA-PK Inhibitor III B07
-0.3˚C, 20 µM PI-103 B08
4-0.1˚C, 20 µM Diacylglycerol Kinase Inhibitor II B09
-0.2˚C, 20 µM DMBI B10
'-0.1˚C, 20 µM EGFR/ErbB-2 Inhibitor B11
-0.6˚C, 0 µM empty B12
0˚C, reference C01
-0.7˚C, 20 µM EGFR Inhibitor C02
.0.1˚C, 20 µM EGFR/ErbB-2/ErbB-4 Inhibitor C03
0.2˚C, 20 µM Flt-3 Inhibitor C04
#0.9˚C, 20 µM Flt-3 Inhibitor II C05
9-0.2˚C, 20 µM cFMS Receptor Tyrosine Kinase Inhibitor C06
0.9˚C, 20 µM Gö 6976 C07
0.4˚C, 20 µM Gö 6983 C08
-0.1˚C, 20 µM GTP-14564 C09
0-0.3˚C, 20 µM Herbimycin A, Streptomyces sp. C10
$0.3˚C, 20 µM Flt-3 Inhibitor III C11
-0.5˚C, 0 µM empty C12
0˚C, reference D01
"0˚C, 20 µM IGF-1R Inhibitor II D02
#0.1˚C, 20 µM IRAK-1/4 Inhibitor D03
1.2˚C, 20 µM JAK Inhibitor I D04
"0.8˚C, 20 µM JAK3 Inhibitor II D05
"0.3˚C, 20 µM JAK3 Inhibitor IV D06
"2.9˚C, 20 µM JAK3 Inhibitor VI D07
-0.2˚C, 20 µM Lck Inhibitor D08
0.8˚C, 20 µM LY 294002 D09
0.1˚C, 20 µM LY 303511 D10
%0.2˚C, 20 µM Met Kinase Inhibitor D11
-0.2˚C, 0 µM empty D12
-0.1˚C, reference E01
0.2˚C, 20 µM PD 158780 E02
0.9˚C, 20 µM PD 174265 E03
<-0.1˚C, 20 µM PDGF Receptor Tyrosine Kinase Inhibitor II E04
:0˚C, 20 µM PDGF Receptor Tyrosine Kinase Inhibitor III E05
;0.3˚C, 20 µM PDGF Receptor Tyrosine Kinase Inhibitor IV E06
$-0.1˚C, 20 µM PDGF RTK Inhibitor E07
2.4˚C, 20 µM PKR Inhibitor E08
00.2˚C, 20 µM PKR Inhibitor, Negative Control E09
#1.4˚C, 10 µM PI 3-Kg Inhibitor E10
&0.2˚C, 20 µM PI 3-Kb Inhibitor II E11
-0.2˚C, 0 µM empty E12
0.2˚C, reference F01
0.6˚C, 20 µM PP3 F02
'0.2˚C, 20 µM PP1 Analog II, 1NM-PP1 F03
'-0.1˚C, 20 µM PKCbII/EGFR Inhibitor F04
0.2˚C, 20 µM PKCb Inhibitor F05
0˚C, 20 µM Rapamycin F06
22.6˚C, 20 µM Rho Kinase Inhibitor III, Rockout F07
)-0.1˚C, 20 µM Rho Kinase Inhibitor IV F08
*1.9˚C, 20 µM Staurosporine, N-benzoyl- F09
(-0.3˚C, 20 µM Src Kinase Inhibitor I F10
1.8˚C, 20 µM SU11652 F11
-0.5˚C, 0 µM empty F12
0˚C, reference G01
0.1˚C, 20 µM Syk Inhibitor G02
!1.2˚C, 20 µM Syk Inhibitor II G03
#-0.2˚C, 20 µM Syk Inhibitor III G04
+-0.3˚C, 20 µM TGF-b RI Kinase Inhibitor G05
(-0.4˚C, 20 µM TGF-b RI Inhibitor III G06
-0.3˚C, 20 µM AG 9 G07
-0.3˚C, 20 µM AG 490 G08
0.4˚C, 20 µM AG 112 G09
-0.2˚C, 20 µM AG 1295 G10
0˚C, 20 µM AG 1296 G11
-0.6˚C, 0 µM empty G12
-0.1˚C, reference H01
0.3˚C, 20 µM AG 1478 H02
30.3˚C, 20 µM VEGF Receptor 2 Kinase Inhibitor I H03
;0.2˚C, 20 µM VEGF Receptor Tyrosine Kinase Inhibitor II H04
E-0.2˚C, 20 µM VEGF Receptor Tyrosine Kinase Inhibitor III, KRN633 H05
20˚C, 20 µM VEGF Receptor 2 Kinase Inhibitor II H06
6-0.2˚C, 20 µM VEGF Receptor 2 Kinase Inhibitor III H07
5-0.1˚C, 20 µM VEGF Receptor 2 Kinase Inhibitor IV H08
$-0.1˚C, 20 µM DNA-PK Inhibitor V H09
--0.4˚C, 20 µM Aurora Kinase Inhibitor III H10
04.4˚C, 20 µM Staurosporine, Streptomyces sp. H11
-0.4˚C, 0 µM empty H12
-2
0
2
4
6
Δ Tm [°C]

Figure S2

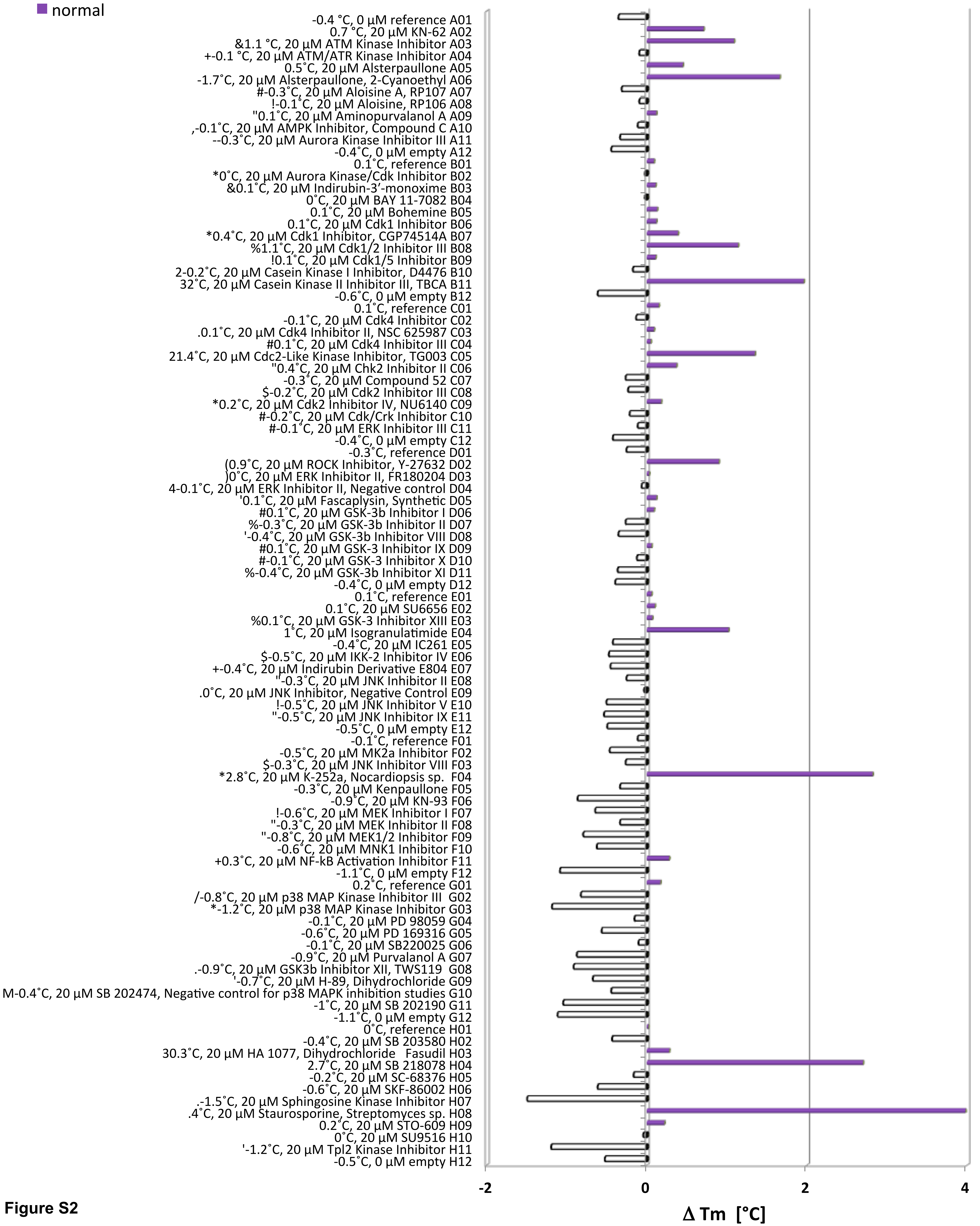
normal
-0.4 °C, 0 µM reference A01
0.7 °C, 20 µM KN-62 A02
&1.1 °C, 20 µM ATM Kinase Inhibitor A03
+-0.1 °C, 20 µM ATM/ATR Kinase Inhibitor A04
0.5˚C, 20 µM Alsterpaullone A05
-1.7˚C, 20 µM Alsterpaullone, 2-Cyanoethyl A06
#-0.3˚C, 20 µM Aloisine A, RP107 A07
!-0.1˚C, 20 µM Aloisine, RP106 A08
"0.1˚C, 20 µM Aminopurvalanol A A09
,-0.1˚C, 20 µM AMPK Inhibitor, Compound C A10
--0.3˚C, 20 µM Aurora Kinase Inhibitor III A11
-0.4˚C, 0 µM empty A12
0.1˚C, reference B01
*0˚C, 20 µM Aurora Kinase/Cdk Inhibitor B02
&0.1˚C, 20 µM Indirubin-3′-monoxime B03
0˚C, 20 µM BAY 11-7082 B04
0.1˚C, 20 µM Bohemine B05
0.1˚C, 20 µM Cdk1 Inhibitor B06
*0.4˚C, 20 µM Cdk1 Inhibitor, CGP74514A B07
%1.1˚C, 20 µM Cdk1/2 Inhibitor III B08
!0.1˚C, 20 µM Cdk1/5 Inhibitor B09
2-0.2˚C, 20 µM Casein Kinase I Inhibitor, D4476 B10
32˚C, 20 µM Casein Kinase II Inhibitor III, TBCA B11
-0.6˚C, 0 µM empty B12
0.1˚C, reference C01
-0.1˚C, 20 µM Cdk4 Inhibitor C02
.0.1˚C, 20 µM Cdk4 Inhibitor II, NSC 625987 C03
#0.1˚C, 20 µM Cdk4 Inhibitor III C04
21.4˚C, 20 µM Cdc2-Like Kinase Inhibitor, TG003 C05
"0.4˚C, 20 µM Chk2 Inhibitor II C06
-0.3˚C, 20 µM Compound 52 C07
$-0.2˚C, 20 µM Cdk2 Inhibitor III C08
*0.2˚C, 20 µM Cdk2 Inhibitor IV, NU6140 C09
#-0.2˚C, 20 µM Cdk/Crk Inhibitor C10
#-0.1˚C, 20 µM ERK Inhibitor III C11
-0.4˚C, 0 µM empty C12
-0.3˚C, reference D01
(0.9˚C, 20 µM ROCK Inhibitor, Y-27632 D02
)0˚C, 20 µM ERK Inhibitor II, FR180204 D03
4-0.1˚C, 20 µM ERK Inhibitor II, Negative control D04
'0.1˚C, 20 µM Fascaplysin, Synthetic D05
#0.1˚C, 20 µM GSK-3b Inhibitor I D06
%-0.3˚C, 20 µM GSK-3b Inhibitor II D07
'-0.4˚C, 20 µM GSK-3b Inhibitor VIII D08
#0.1˚C, 20 µM GSK-3 Inhibitor IX D09
#-0.1˚C, 20 µM GSK-3 Inhibitor X D10
%-0.4˚C, 20 µM GSK-3b Inhibitor XI D11
-0.4˚C, 0 µM empty D12
0.1˚C, reference E01
0.1˚C, 20 µM SU6656 E02
%0.1˚C, 20 µM GSK-3 Inhibitor XIII E03
1˚C, 20 µM Isogranulatimide E04
-0.4˚C, 20 µM IC261 E05
$-0.5˚C, 20 µM IKK-2 Inhibitor IV E06
+-0.4˚C, 20 µM Indirubin Derivative E804 E07
"-0.3˚C, 20 µM JNK Inhibitor II E08
.0˚C, 20 µM JNK Inhibitor, Negative Control E09
!-0.5˚C, 20 µM JNK Inhibitor V E10
"-0.5˚C, 20 µM JNK Inhibitor IX E11
-0.5˚C, 0 µM empty E12
-0.1˚C, reference F01
-0.5˚C, 20 µM MK2a Inhibitor F02
$-0.3˚C, 20 µM JNK Inhibitor VIII F03
*2.8˚C, 20 µM K-252a, Nocardiopsis sp. F04
-0.3˚C, 20 µM Kenpaullone F05
-0.9˚C, 20 µM KN-93 F06
!-0.6˚C, 20 µM MEK Inhibitor I F07
"-0.3˚C, 20 µM MEK Inhibitor II F08
"-0.8˚C, 20 µM MEK1/2 Inhibitor F09
-0.6˚C, 20 µM MNK1 Inhibitor F10
+0.3˚C, 20 µM NF-kB Activation Inhibitor F11
-1.1˚C, 0 µM empty F12
0.2˚C, reference G01
/-0.8˚C, 20 µM p38 MAP Kinase Inhibitor III G02
*-1.2˚C, 20 µM p38 MAP Kinase Inhibitor G03
-0.1˚C, 20 µM PD 98059 G04
-0.6˚C, 20 µM PD 169316 G05
-0.1˚C, 20 µM SB220025 G06
-0.9˚C, 20 µM Purvalanol A G07
.-0.9˚C, 20 µM GSK3b Inhibitor XII, TWS119 G08
'-0.7˚C, 20 µM H-89, Dihydrochloride G09
M-0.4˚C, 20 µM SB 202474, Negative control for p38 MAPK inhibition studies G10
-1˚C, 20 µM SB 202190 G11
-1.1˚C, 0 µM empty G12
0˚C, reference H01
-0.4˚C, 20 µM SB 203580 H02
30.3˚C, 20 µM HA 1077, Dihydrochloride Fasudil H03
2.7˚C, 20 µM SB 218078 H04
-0.2˚C, 20 µM SC-68376 H05
-0.6˚C, 20 µM SKF-86002 H06
.-1.5˚C, 20 µM Sphingosine Kinase Inhibitor H07
.4˚C, 20 µM Staurosporine, Streptomyces sp. H08
0.2˚C, 20 µM STO-609 H09
0˚C, 20 µM SU9516 H10
'-1.2˚C, 20 µM Tpl2 Kinase Inhibitor H11
-0.5˚C, 0 µM empty H12
-2
0
2
4
Δ Tm [°C]

Figure S2

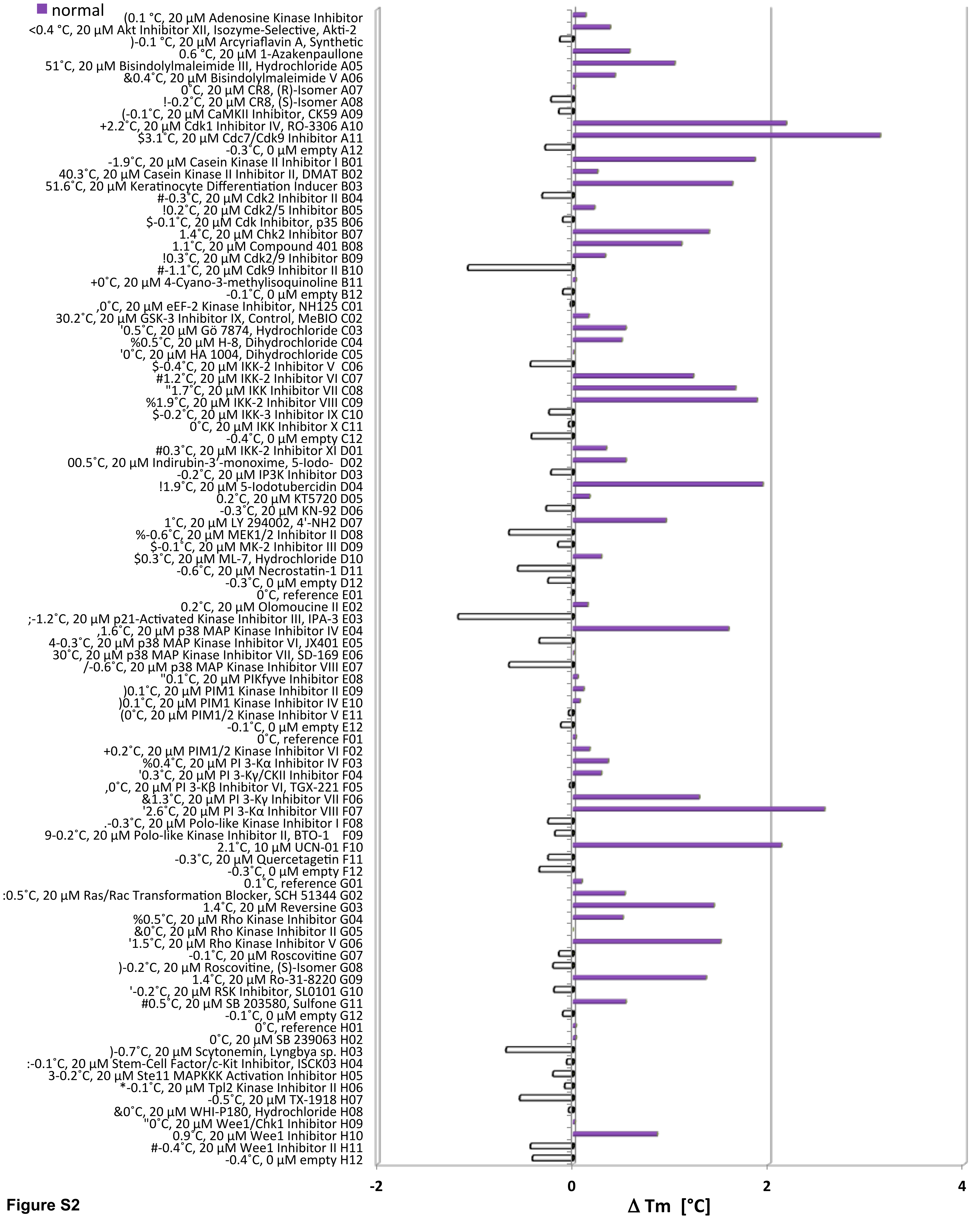
normal
(0.1 °C, 20 µM Adenosine Kinase Inhibitor
<0.4 °C, 20 µM Akt Inhibitor XII, Isozyme-Selective, Akti-2
)-0.1 °C, 20 µM Arcyriaflavin A, Synthetic
0.6 °C, 20 µM 1-Azakenpaullone
51˚C, 20 µM Bisindolylmaleimide III, Hydrochloride A05
&0.4˚C, 20 µM Bisindolylmaleimide V A06
0˚C, 20 µM CR8, (R)-Isomer A07
!-0.2˚C, 20 µM CR8, (S)-Isomer A08
(-0.1˚C, 20 µM CaMKII Inhibitor, CK59 A09
+2.2˚C, 20 µM Cdk1 Inhibitor IV, RO-3306 A10
$3.1˚C, 20 µM Cdc7/Cdk9 Inhibitor A11
-0.3˚C, 0 µM empty A12
-1.9˚C, 20 µM Casein Kinase II Inhibitor I B01
40.3˚C, 20 µM Casein Kinase II Inhibitor II, DMAT B02
51.6˚C, 20 µM Keratinocyte Differentiation Inducer B03
#-0.3˚C, 20 µM Cdk2 Inhibitor II B04
!0.2˚C, 20 µM Cdk2/5 Inhibitor B05
$-0.1˚C, 20 µM Cdk Inhibitor, p35 B06
1.4˚C, 20 µM Chk2 Inhibitor B07
1.1˚C, 20 µM Compound 401 B08
!0.3˚C, 20 µM Cdk2/9 Inhibitor B09
#-1.1˚C, 20 µM Cdk9 Inhibitor II B10
+0˚C, 20 µM 4-Cyano-3-methylisoquinoline B11
-0.1˚C, 0 µM empty B12
,0˚C, 20 µM eEF-2 Kinase Inhibitor, NH125 C01
30.2˚C, 20 µM GSK-3 Inhibitor IX, Control, MeBIO C02
'0.5˚C, 20 µM Gö 7874, Hydrochloride C03
%0.5˚C, 20 µM H-8, Dihydrochloride C04
'0˚C, 20 µM HA 1004, Dihydrochloride C05
$-0.4˚C, 20 µM IKK-2 Inhibitor V C06
#1.2˚C, 20 µM IKK-2 Inhibitor VI C07
"1.7˚C, 20 µM IKK Inhibitor VII C08
%1.9˚C, 20 µM IKK-2 Inhibitor VIII C09
$-0.2˚C, 20 µM IKK-3 Inhibitor IX C10
0˚C, 20 µM IKK Inhibitor X C11
-0.4˚C, 0 µM empty C12
#0.3˚C, 20 µM IKK-2 Inhibitor XI D01
00.5˚C, 20 µM Indirubin-3′-monoxime, 5-Iodo- D02
-0.2˚C, 20 µM IP3K Inhibitor D03
!1.9˚C, 20 µM 5-Iodotubercidin D04
0.2˚C, 20 µM KT5720 D05
-0.3˚C, 20 µM KN-92 D06
1˚C, 20 µM LY 294002, 4'-NH2 D07
%-0.6˚C, 20 µM MEK1/2 Inhibitor II D08
$-0.1˚C, 20 µM MK-2 Inhibitor III D09
$0.3˚C, 20 µM ML-7, Hydrochloride D10
-0.6˚C, 20 µM Necrostatin-1 D11
-0.3˚C, 0 µM empty D12
0˚C, reference E01
0.2˚C, 20 µM Olomoucine II E02
;-1.2˚C, 20 µM p21-Activated Kinase Inhibitor III, IPA-3 E03
,1.6˚C, 20 µM p38 MAP Kinase Inhibitor IV E04
4-0.3˚C, 20 µM p38 MAP Kinase Inhibitor VI, JX401 E05
30˚C, 20 µM p38 MAP Kinase Inhibitor VII, SD-169 E06
/-0.6˚C, 20 µM p38 MAP Kinase Inhibitor VIII E07
"0.1˚C, 20 µM PIKfyve Inhibitor E08
)0.1˚C, 20 µM PIM1 Kinase Inhibitor II E09
)0.1˚C, 20 µM PIM1 Kinase Inhibitor IV E10
(0˚C, 20 µM PIM1/2 Kinase Inhibitor V E11
-0.1˚C, 0 µM empty E12
0˚C, reference F01
+0.2˚C, 20 µM PIM1/2 Kinase Inhibitor VI F02
%0.4˚C, 20 µM PI 3-Kα Inhibitor IV F03
'0.3˚C, 20 µM PI 3-Kγ/CKII Inhibitor F04
,0˚C, 20 µM PI 3-Kβ Inhibitor VI, TGX-221 F05
&1.3˚C, 20 µM PI 3-Kγ Inhibitor VII F06
'2.6˚C, 20 µM PI 3-Kα Inhibitor VIII F07
.-0.3˚C, 20 µM Polo-like Kinase Inhibitor I F08
9-0.2˚C, 20 µM Polo-like Kinase Inhibitor II, BTO-1 F09
2.1˚C, 10 µM UCN-01 F10
-0.3˚C, 20 µM Quercetagetin F11
-0.3˚C, 0 µM empty F12
0.1˚C, reference G01
:0.5˚C, 20 µM Ras/Rac Transformation Blocker, SCH 51344 G02
1.4˚C, 20 µM Reversine G03
%0.5˚C, 20 µM Rho Kinase Inhibitor G04
&0˚C, 20 µM Rho Kinase Inhibitor II G05
'1.5˚C, 20 µM Rho Kinase Inhibitor V G06
-0.1˚C, 20 µM Roscovitine G07
)-0.2˚C, 20 µM Roscovitine, (S)-Isomer G08
1.4˚C, 20 µM Ro-31-8220 G09
'-0.2˚C, 20 µM RSK Inhibitor, SL0101 G10
#0.5˚C, 20 µM SB 203580, Sulfone G11
-0.1˚C, 0 µM empty G12
0˚C, reference H01
0˚C, 20 µM SB 239063 H02
)-0.7˚C, 20 µM Scytonemin, Lyngbya sp. H03
:-0.1˚C, 20 µM Stem-Cell Factor/c-Kit Inhibitor, ISCK03 H04
3-0.2˚C, 20 µM Ste11 MAPKKK Activation Inhibitor H05
*-0.1˚C, 20 µM Tpl2 Kinase Inhibitor II H06
-0.5˚C, 20 µM TX-1918 H07
&0˚C, 20 µM WHI-P180, Hydrochloride H08
"0˚C, 20 µM Wee1/Chk1 Inhibitor H09
0.9˚C, 20 µM Wee1 Inhibitor H10
#-0.4˚C, 20 µM Wee1 Inhibitor II H11
-0.4˚C, 0 µM empty H12
-2
0
2
4
Δ Tm [°C]

Figure S2

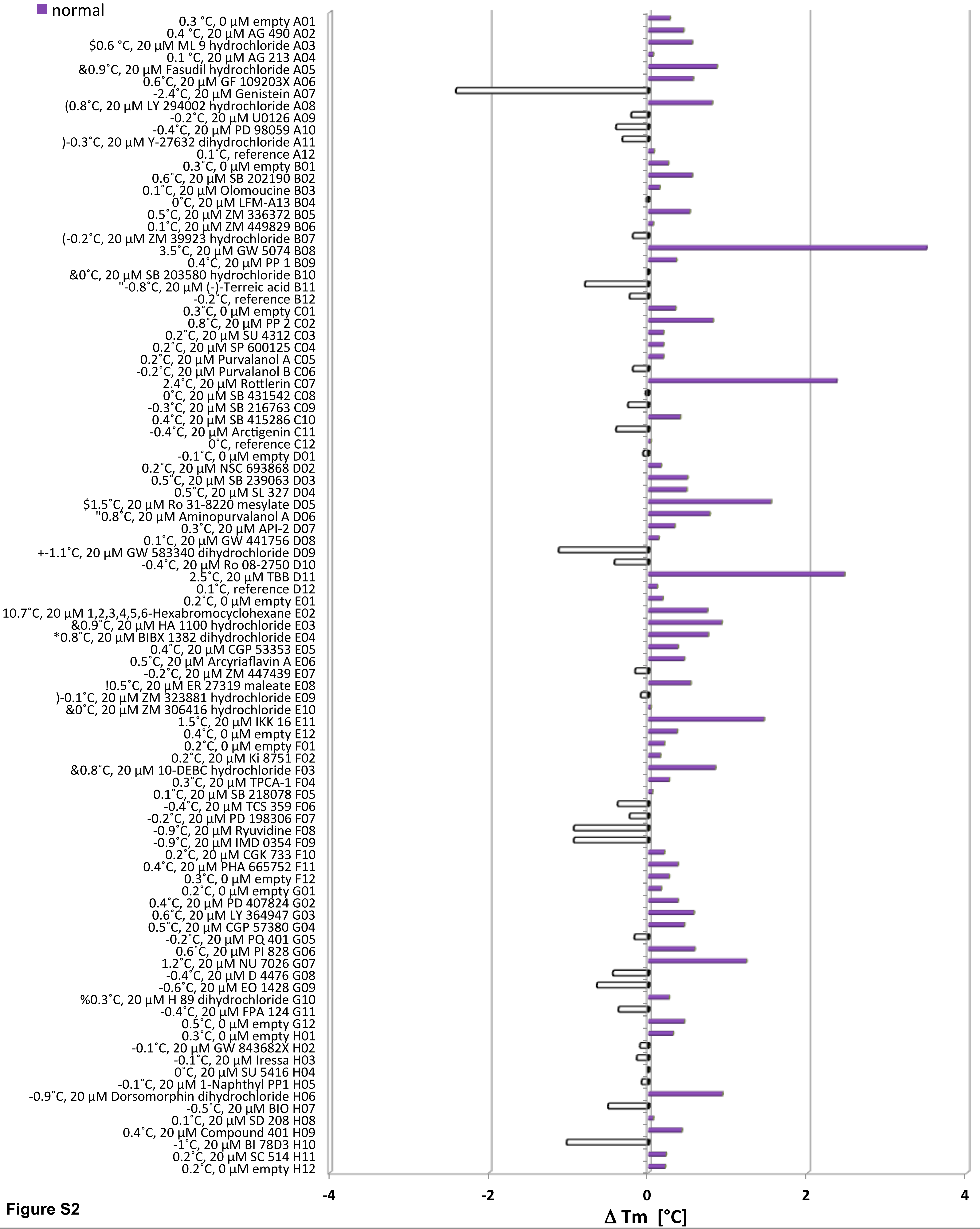
normal
0.3 °C, 0 µM empty A01
0.4 °C, 20 µM AG 490 A02
$0.6 °C, 20 µM ML 9 hydrochloride A03
0.1 °C, 20 µM AG 213 A04
&0.9˚C, 20 µM Fasudil hydrochloride A05
0.6˚C, 20 µM GF 109203X A06
-2.4˚C, 20 µM Genistein A07
(0.8˚C, 20 µM LY 294002 hydrochloride A08
-0.2˚C, 20 µM U0126 A09
-0.4˚C, 20 µM PD 98059 A10
)-0.3˚C, 20 µM Y-27632 dihydrochloride A11
0.1˚C, reference A12
0.3˚C, 0 µM empty B01
0.6˚C, 20 µM SB 202190 B02
0.1˚C, 20 µM Olomoucine B03
0˚C, 20 µM LFM-A13 B04
0.5˚C, 20 µM ZM 336372 B05
0.1˚C, 20 µM ZM 449829 B06
(-0.2˚C, 20 µM ZM 39923 hydrochloride B07
3.5˚C, 20 µM GW 5074 B08
0.4˚C, 20 µM PP 1 B09
&0˚C, 20 µM SB 203580 hydrochloride B10
"-0.8˚C, 20 µM (-)-Terreic acid B11
-0.2˚C, reference B12
0.3˚C, 0 µM empty C01
0.8˚C, 20 µM PP 2 C02
0.2˚C, 20 µM SU 4312 C03
0.2˚C, 20 µM SP 600125 C04
0.2˚C, 20 µM Purvalanol A C05
-0.2˚C, 20 µM Purvalanol B C06
2.4˚C, 20 µM Rottlerin C07
0˚C, 20 µM SB 431542 C08
-0.3˚C, 20 µM SB 216763 C09
0.4˚C, 20 µM SB 415286 C10
-0.4˚C, 20 µM Arctigenin C11
0˚C, reference C12
-0.1˚C, 0 µM empty D01
0.2˚C, 20 µM NSC 693868 D02
0.5˚C, 20 µM SB 239063 D03
0.5˚C, 20 µM SL 327 D04
$1.5˚C, 20 µM Ro 31-8220 mesylate D05
"0.8˚C, 20 µM Aminopurvalanol A D06
0.3˚C, 20 µM API-2 D07
0.1˚C, 20 µM GW 441756 D08
+-1.1˚C, 20 µM GW 583340 dihydrochloride D09
-0.4˚C, 20 µM Ro 08-2750 D10
2.5˚C, 20 µM TBB D11
0.1˚C, reference D12
0.2˚C, 0 µM empty E01
10.7˚C, 20 µM 1,2,3,4,5,6-Hexabromocyclohexane E02
&0.9˚C, 20 µM HA 1100 hydrochloride E03
*0.8˚C, 20 µM BIBX 1382 dihydrochloride E04
0.4˚C, 20 µM CGP 53353 E05
0.5˚C, 20 µM Arcyriaflavin A E06
-0.2˚C, 20 µM ZM 447439 E07
!0.5˚C, 20 µM ER 27319 maleate E08
)-0.1˚C, 20 µM ZM 323881 hydrochloride E09
&0˚C, 20 µM ZM 306416 hydrochloride E10
1.5˚C, 20 µM IKK 16 E11
0.4˚C, 0 µM empty E12
0.2˚C, 0 µM empty F01
0.2˚C, 20 µM Ki 8751 F02
&0.8˚C, 20 µM 10-DEBC hydrochloride F03
0.3˚C, 20 µM TPCA-1 F04
0.1˚C, 20 µM SB 218078 F05
-0.4˚C, 20 µM TCS 359 F06
-0.2˚C, 20 µM PD 198306 F07
-0.9˚C, 20 µM Ryuvidine F08
-0.9˚C, 20 µM IMD 0354 F09
0.2˚C, 20 µM CGK 733 F10
0.4˚C, 20 µM PHA 665752 F11
0.3˚C, 0 µM empty F12
0.2˚C, 0 µM empty G01
0.4˚C, 20 µM PD 407824 G02
0.6˚C, 20 µM LY 364947 G03
0.5˚C, 20 µM CGP 57380 G04
-0.2˚C, 20 µM PQ 401 G05
0.6˚C, 20 µM PI 828 G06
1.2˚C, 20 µM NU 7026 G07
-0.4˚C, 20 µM D 4476 G08
-0.6˚C, 20 µM EO 1428 G09
%0.3˚C, 20 µM H 89 dihydrochloride G10
-0.4˚C, 20 µM FPA 124 G11
0.5˚C, 0 µM empty G12
0.3˚C, 0 µM empty H01
-0.1˚C, 20 µM GW 843682X H02
-0.1˚C, 20 µM Iressa H03
0˚C, 20 µM SU 5416 H04
-0.1˚C, 20 µM 1-Naphthyl PP1 H05
-0.9˚C, 20 µM Dorsomorphin dihydrochloride H06
-0.5˚C, 20 µM BIO H07
0.1˚C, 20 µM SD 208 H08
0.4˚C, 20 µM Compound 401 H09
-1˚C, 20 µM BI 78D3 H10
0.2˚C, 20 µM SC 514 H11
0.2˚C, 0 µM empty H12
-4
-2
0
2
4
Δ Tm [°C]

Figure S2

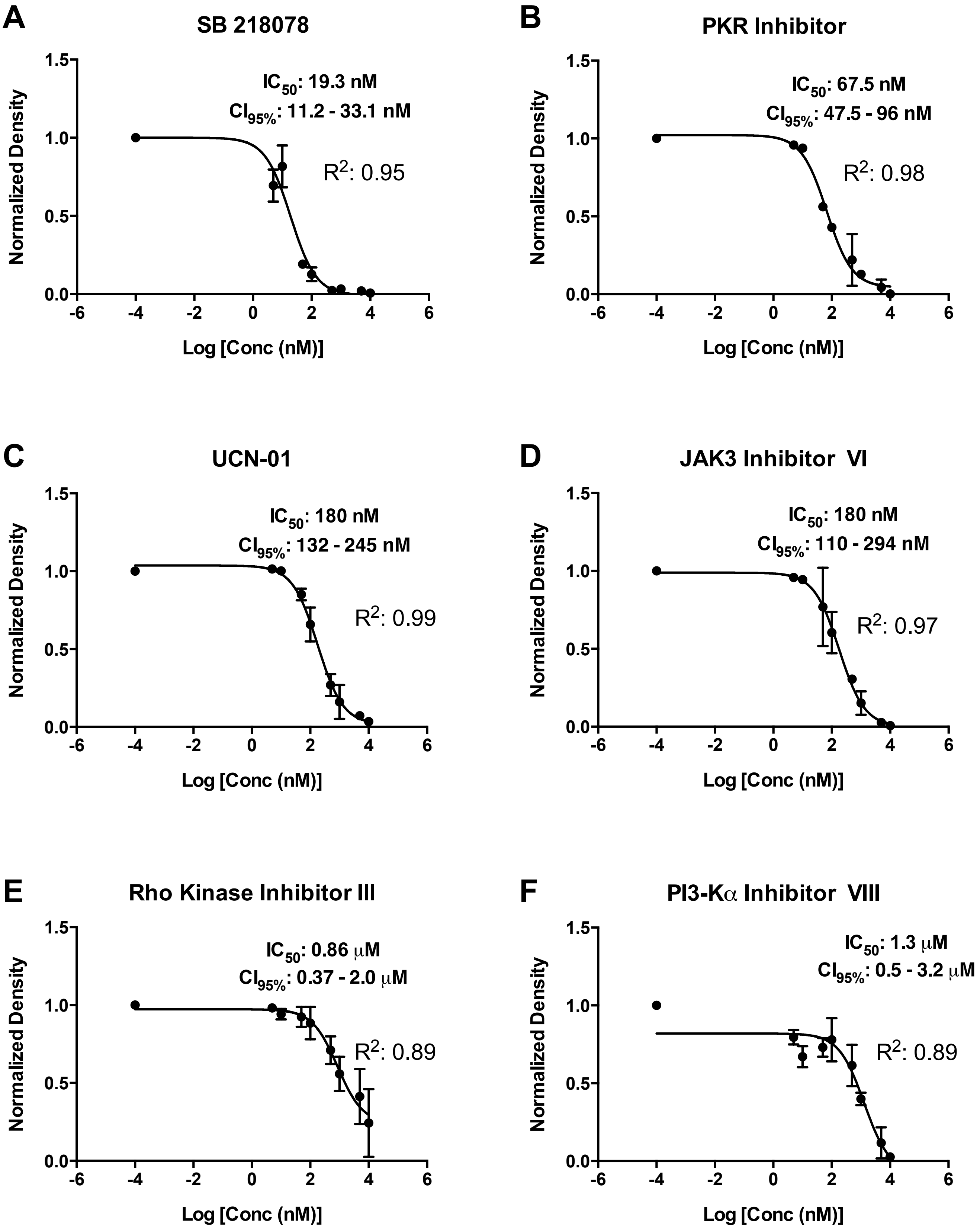

A
SB 218078
IC50: 19.3 nM
CI95%: 11.2 - 33.1 nM
R2: 0.95
B
PKR Inhibitor
IC50: 67.5 nM
CI95%: 47.5 - 96 nM
R2: 0.98
C
UCN-01
IC50: 180 nM
CI95%: 132 - 245 nM
R2: 0.99
D
JAK3 Inhibitor VI
IC50: 180 nM
CI95%: 110 - 294 nM
R2: 0.97
E
Rho Kinase Inhibitor III
IC50: 0.86 μM
CI95%: 0.37 - 2.0 μM
R2: 0.89
F
PI3-Kα Inhibitor VIII
IC50: 1.3 μM
CI95%: 0.5 - 3.2 μM
R2: 0.89
Normalized Density
Log [Conc (nM)]

Figure S3

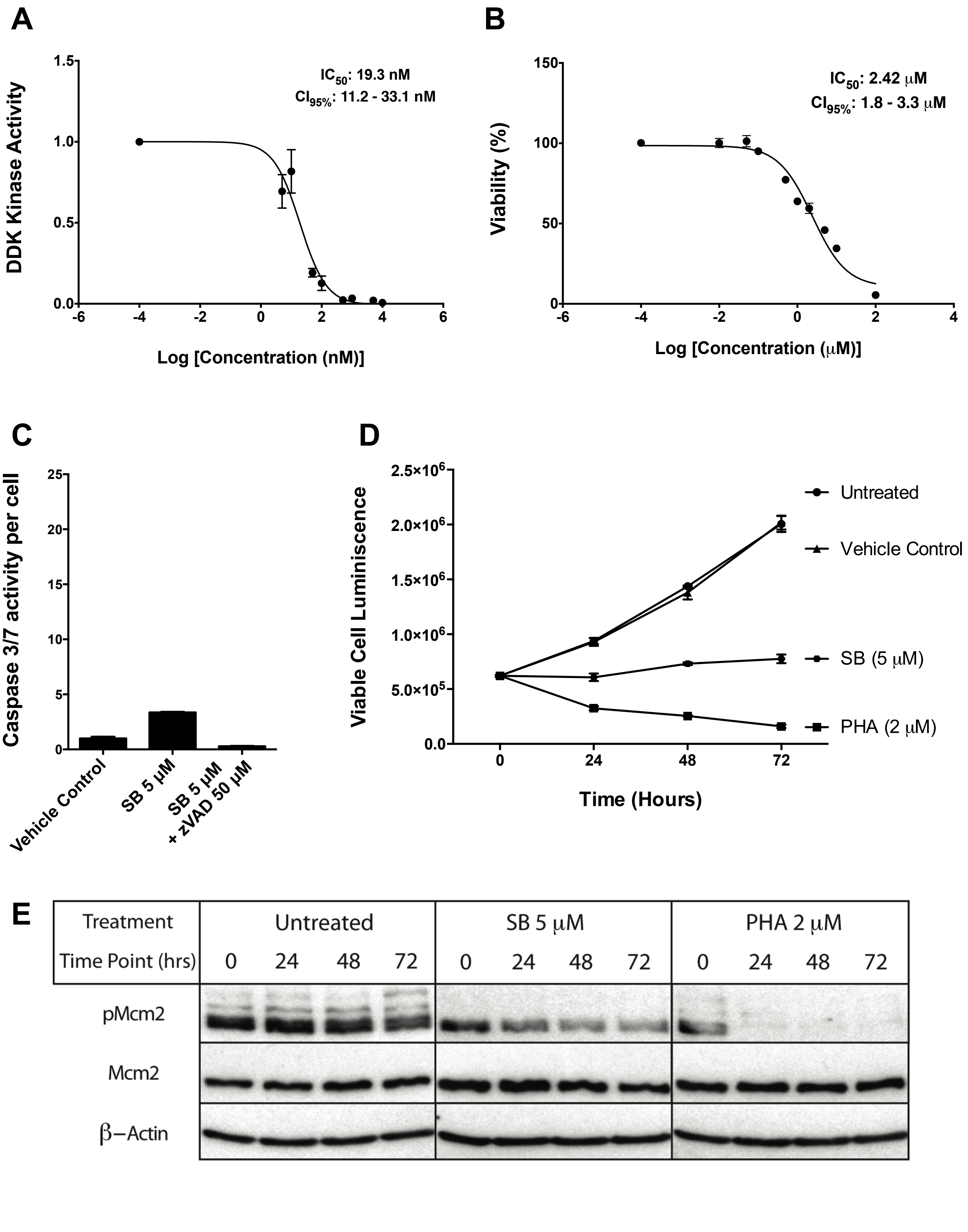
A
DDK Kinase Activity
Log [Concentration (nM)]
IC50: 19.3 nM
CI95%: 11.2 - 33.1 nM
B
Viability (%)
Log [Concentration (μM)]
IC50: 2.42 μM
CI95%: 1.8 - 3.3 μM
C
Caspase 3/7 activity per cell
Vehicle Control
SB 5 μM
SB 5 μM + zVAD 50 μM
D
Viable Cell Luminiscence
Time (Hours)
Untreated
Vehicle Control
SB (5 μM)
PHA (2 μM)
E
Treatment
Time Point (hrs)
Untreated
SB 5 μM
PHA 2 μM
0 24 48 72
pMcm2
Mcm2
β−Actin

Figure S4

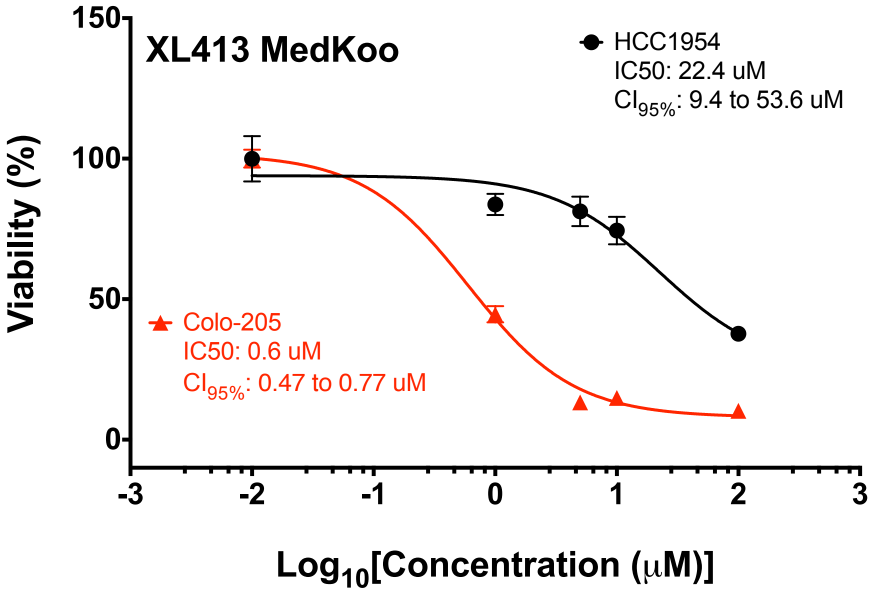

XL413 MedKoo
HCC1954
IC50: 22.4 uM
CI95%: 9.4 to 53.6 uM
Colo-205
IC50: 0.6 uM
CI95%: 0.47 to 0.77 uM
Viability (%)
150
100
50
0
-3
-2
-1
0
1
2
3
Log10[Concentration (μM)]

Figure S5

TABLE S1 – Characteristics of top kinase inhibitors discovered in TSA screen

Structure	Description	IUPAC Name	Molecular Weight	Vendor/ Catalogue Number
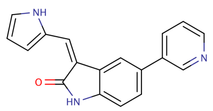	JAK3 Inhibitor VI	methanesulfonic acid;(3Z)-5-pyridin-3-yl-3-(1H-pyrrol-2-ylmethylidene)-1H-indol-2-one	383.4	Calbiochem/ 420126
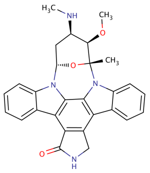	Staurosporine (Broad spectrum protein kinase inhibitor - PKA, PKC, CAMK II etc.)	Staurosporine	466.5	Calbiochem/ 569397
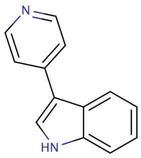	Rockout, Rho Kinase Inhibitor III	3-pyridin-4-yl-1H-indole	194.2	Calbiochem/ 555553
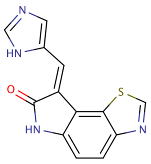	PKR Inhibitor	(8Z)-8-(1H-imidazol-5-ylmethylidene)-6H-pyrrolo[2,3-g][1,3] benzothiazol-7-one	268.3	Calbiochem/ 527450
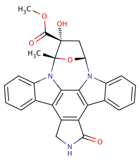	K-252a (Broad spectrum protein kinase inhibitor - PKA, PKC, Cdc2, Cdc25 etc.)	Staurosporine agylcone	467.5	Calbiochem/ 420298
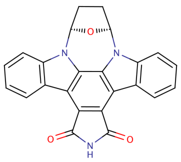	SB 218078 (Chk1 Inhibitor)	Staurosporine derivative	393.4	Calbiochem/ 559402
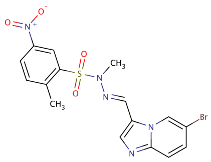	PI 3-Kα Inhibitor VIII	N- ((1E)-(6-Bromoimidazo[1,2-a]pyridin-3-yl)methylene)-N'-methyl-N''-(2-methyl-5-nitrobenzene)sulfonohydrazide, HCl	524.8	Calbiochem/ 528116

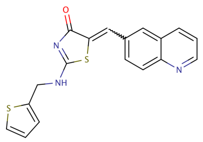	RO-3306, (CDK1 Inhibitor IV)	5-(6-Quinolinylmethylene)-2-[(2-thienylmethyl) amino]-4(5*H*)-thiazolone	351.5	Calbiochem/ 217699
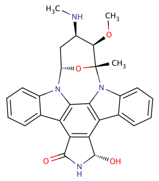	UCN-01 (Broad spectrum protein kinase inhibitor – PKC, Chk1, Cdk1 etc.)	7-Hydroxystaurosporine	482.5	Calbiochem/ 539644
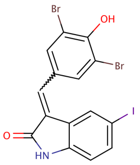	GW 5074 (c-RAF1 inhibitor)	3-(3,5-Dibromo-4-hydroxy-benzylidene)-5-iodo-1, 3-dihydro-indol-2-one	520.94	TOCRIS/ 1381
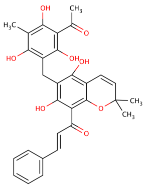	Rottlerin (PKC Inhibitor)	3'-[(8-Cinnamoyl-5,7-dihydroxy-2,2-dimethyl-2H-1-benzopyran-6-yl)methyl]-2',4',6'-trihydroxy-5'-methylacetophenone	516.55	TOCRIS/ 1610
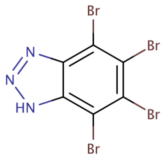	TBB (CK2 Inhibitor)	4,5,6,7-Tetrabromobenzotriazole	434.71	TOCRIS/ 2275
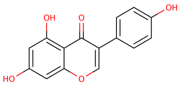	Genistein (EGFR Inhibitor)	5,7-Dihydroxy-3-(4-hydroxyphenyl)-4H-1-benzopyran-4-one	270.24	TOCRIS/ 1110
